# Exosomes derived from adipose-derived stem cells alleviate acute radiation-induced dermatitis through up-regulating hyaluronic acid synthase 1 expression

**DOI:** 10.1186/s13287-025-04276-8

**Published:** 2025-05-20

**Authors:** Meijia Li, Yuan Tian, Xiaotian Wang, Di Sun, Haiqian Xu, Xinyue Wang, Xinyue Chen, Lijun Hao

**Affiliations:** 1 The Center of Plastic and Aesthetic Surgery of the First Affiliated Hospital of Harbin Medical University, Harbin, 150001 People’s Republic of China; 2 The Department of Radiology of the Cancer Hospital of Harbin Medical University, Harbin, People’s Republic of China

**Keywords:** Adipose-derived stem cell exosomes, Hyaluronic acid, Hyaluronic acid synthase, Acute radiation-induced dermatitis, Mesenchymal stem cells

## Abstract

**Background:**

Acute radiation-induced dermatitis refers to skin lesions that usually appear within 90 days of the start of radiotherapy. Although various treatments are available, none have proven fully effective. Exosomes produced by adipose-derived stem cells play crucial roles in enhancing cell regeneration, promoting angiogenesis, regulating inflammation and remodeling the extracellular matrix. Hyaluronic acid, a major extracellular matrix component, is synthesized by hyaluronic acid synthase, with hyaluronic acid synthase 1 being particularly critical for skin repair. This study aimed to investigate whether exosomes derived from adipose-derived stem cells can protect against radiation-induced acute skin damage and to elucidate the underlying mechanisms involving hyaluronic acid synthase 1.

**Methods:**

Thirty-six male adult SD rats were randomly divided into a negative control group, an irradiation group (90 Gy), and a radiation + exosomes group (90 Gy + 100 ug exosomes). Three groups of fibroblasts were assigned: one for control, one for radiation (6 Gy), and one for radiation plus exosomes (6 Gy + 4 ug exosomes). The effect of ADSC-exos transplantation was evaluated using skin damage score, histopathological analysis, electron microscopy, immunohistochemical staining, immunofluorescence staining, and immunoblotting analysis. Furthermore, small interfering RNA-mediated knockdown of hyaluronic acid synthase 1 was performed to explore its regulatory role in the TGF-β/Smad2/3 signaling pathway.

**Results:**

After irradiation, the ADSC-exos intervention significantly increased the levels of stromal cell-derived factor-1, matrix metalloproteinases, transforming growth factor, basic fibroblast growth factor, platelet-derived growth factor, vascular endothelial growth factor, interleukin 10, interleukin 12, and reduced the expression of the pro-inflammatory factor interleukin 6. Notably, exosomes treatment markedly upregulated hyaluronic acid synthase 1 expression, and small interfering RNA-mediated knockdown of hyaluronic acid synthase 1 resulted in reduced phosphorylation of TGF-β/Smad2/3 signaling components, indicating that hyaluronic acid synthase 1 is a critical mediator of this pathway.

**Conclusion:**

Exosomes derived from adipose-derived stem cells alleviate acute radiation-induced dermatitis by enhancing hyaluronic acid synthase 1 expression and activating the TGF-β/Smad2/3 pathway, thereby promoting skin regeneration and repair. These findings suggest that exosomes derived from adipose-derived stem cells may serve as a promising cell-free therapeutic strategy for the prevention and treatment of acute radiation-induced dermatitis.

**Graphical abstract:**

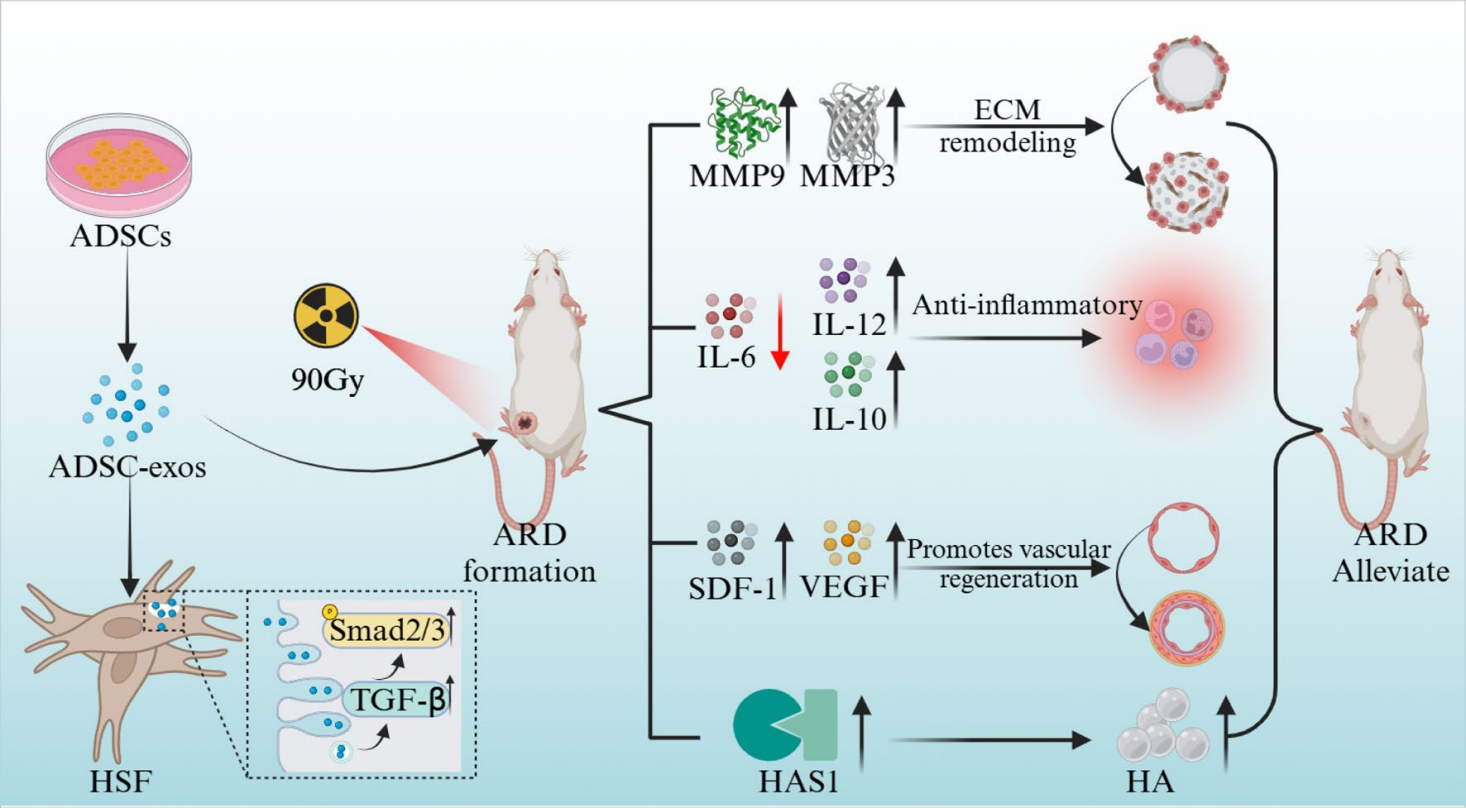

**Supplementary Information:**

The online version contains supplementary material available at 10.1186/s13287-025-04276-8.

## Background

There are many types of organ damage caused by radiation, including bone marrow, brain, intestines, lungs, salivary glands, etc. [1–5]. Skin, like bone marrow, is particularly sensitive to ionizing radiation, with up to 95% of patients developing moderate to severe reactions. Currently, topical medications used to treat radiation-induced skin damage include glucocorticoids, sucralfate, and silver ion dressings [6], though a standardized treatment protocol has yet to be established. Acute radiation-induced dermatitis(ARD) usually appears a few days after treatment, with redness and skin peeling. Chronic radiation-induced dermatitis(CRD) is characterized by skin fibrosis and thickness after 90 days of irradiation [7]. CRD models are often accompanied by severe skin fibrosis, and common drug treatments are less effective. Surgical treatment is often required in clinical practice. Thus, the primary emphasis of this investigation is acute radiation skin injury.

Mesenchymal stem cells (MSCs) are effective treatments for tissue engineering, autoimmune diseases, and graft-versus-host disease [8]. Previous research have demonstrated that bone marrow mesenchymal stem cells can prevent radiation-induced liver damage [9], increase wound healing for radiation-induced skin damage [10–12], and improve the longevity of irradiated animals [13]. Human umbilical cord mesenchymal stem cells have been demonstrated to promote the healing of irradiation-induced skin ulcers by increasing keratin synthesis and epithelial cell proliferation [14]. The secretome of human fetal mesenchymal stem cells accelerates the rate of wound healing in rats with radiation-induced skin damage by promoting angiogenesis [15]. Several studies have shown that stem cell-based interventions can effectively alleviate and repair radiation-related damage [16]. Among MSCs, Adipose-derived stem cells (ADSCs) are increasingly favored due to their abundance, ease of isolation, and strong regenerative capabilities. They play a key role in skin and soft tissue regeneration and have been shown to promote angiogenesis, modulate inflammation, enhance extracellular matrix remodeling, and exhibit potent antioxidant activity [17]. However, during the past several years, an increasing amount of research has shown that paracrine pathways, particularly those involving exosomes, are the primary means by which mesenchymal stem cells aid healing. Exosomes are microscopic vesicles with a diameter of 40–100 nm that are surrounded by a bio-lipid membrane. They function as extracellular messengers by transporting their contents, such as microRNAs (miRNAs), messenger RNAs (mRNAs), cell adhesion molecules, cytokines, and proteins [18]. As opposed to ADSCs, exosome-based acellular treatment offers numerous benefits, including the capacity to cross the blood–brain barrier, low immunogenicity, low tumorigenicity, low cytotoxicity, easy storage, high stability, low degradation, and potential intravenous administration. ADSCs are less impacted by age and skeletal circumstances than bone marrow mesenchymal stem cells [19]. Thus, we decided to use ADSCs as the exosome source in this investigation.

One of the most prevalent and abundant glycosaminoglycans in the extracellular matrix is hyaluronic acid (HA). It participates in biological processes such as cell growth and migration serving as a core component of the ECM [20]. It is crucial for processes like embryonic development, wound healing, inflammation, and the growth of malignancies [21, 22]. Moreover, HA has shown remarkable results in the treatment of radioactive skin damage [23, 24]. The hyaluronan synthase family, including HAS1, HAS2, and HAS3, synthesizes HA [25, 26]. Studies have shown that the loss of hyaluronan synthase can affect the shape, quality and mechanical properties of bone [27]. Other studies have shown that the selective deletion of HAS1 and HAS3 can increase inflammation in the dermis [28]. Among them, HAS1 is especially expressed in fibroblasts and plays a vital role in skin function [29]. In recent years, scholars have published relevant literature in NATURE and SCIENCE magazines, finding that mice overexpressing the HAS2 gene exhibit a reduced incidence of both spontaneous and induced cancers, an extended life expectancy, and improved overall health [30]. Removing the trimethylated H3K27 (H3K27me3) mark on the HAS2 site can initiate HA production, exert pro-regenerative effects and accelerate the inflammatory repair of muscle stem cells and muscle repair [31]. Therefore, this study will explore the effect of ADSC-exos in preventing acute radiation-induced skin damage while also investigating its mechanism of action with HAS.

## Materials and methods

### Extraction and characterization of ADSCs

The work has been reported according to the ARRIVE guidelines 2.0. Adipose tissue was obtained from donors who underwent elective liposuction at the First Hospital of Harbin Medical University. The collected tissues were washed extensively with PBS containing 1% penicillin–streptomycin to remove blood and debris. Then, the tissues were enzymatically digested with 0.1% type I collagenase (Sigma-Aldrich, USA) at 37 °C with gentle agitation for 45 min. The enzymatic activity was neutralized by adding an equal volume of DMEM supplemented with 10% FBS. The resulting cell suspension was filtered through a 100-μm mesh to remove undigested debris and then centrifuged at 300 × g rpm for 5 min to precipitate the stromal vascular fraction. The cell pellet was resuspended in DMEM containing 10% FBS, 1% penicillin–streptomycin, and incubated in T75 flasks humidified with 5% CO_2_ at 37 °C. The medium was replaced every 2–3 days. The medium was changed every 2–3 days, and when the cells reached 80–90% confluence, the adherent cells were detached with 0.25% trypsin and then passaged for expansion.

ADSCs are known for their tri-lineage differentiation potential (osteogenic, adipogenic, and chondrogenic). In this study, we focused on osteogenic and adipogenic differentiation, which are more directly related to the regenerative functions of ADSCs in the context of radiation-induced skin injury. Standard differentiation protocols were used for osteogenic (Alizarin Red S staining) and adipogenic (Oil Red O staining) differentiation. Flow cytometry was used to analyze the immunophenotype of ADSCs, including the expression of CD29, CD34, CD44, CD45, CD14, and CD105.

### Preparation of ADSC-exos

The fourth-generation ADSCs were cultured for an additional 24–48 h at a density of 80–90% in a medium containing 10% exosome-depleted FBS and no extracellular vesicles (Gibco, USA). The supernatant of collected ADSCs was centrifuged, filtered, and transferred to an exosome high-permeability filter tube. The tube was mounted on a T32S5 rotor and moved to an ultracentrifuge, spinning at 100,000 rpm for 70 min. The supernatant was collected, and the precipitate was resuspended in PBS before being ultracentrifuged at 100,000 rpm for 70 min at 4 °C.The supernatant was removed, leaving just the ADSC-exos precipitate. The protein concentration was determined using a BCA protein assay kit. The exosomes were mixed with 100 μl of PBS to achieve a concentration of approximately 0.5 μg/μl. An electron microscope was used to examine the size and morphological makeup of exosomes. Nanoparticle tracking (Nano Sight NS300) was used to measure the size of isolated exosomes. The levels of CD63, CD9 and TSG101 proteins in both ADSCs and ADSC-exos were evaluated using western blot. ADSC-exos were co-cultured with HSF for 24 h after being labeled with the PKH67 fluorescent cell linker kit (Sigma-Aldrich, MIDI67-1KT). The nuclei were stained with DAPI (Solarbio, C0065), and images were obtained under an Olympus IX81 fluorescence microscope to confirm that ADSC-exos were internalized by fibroblasts. The collected ADSC-exos were either used right away for additional research or frozen at − 80 °C.

### Construction of radioactive animal model

We purchased thirty-six 220–250 g of Sprague–Dawley (SD) rats from Harbin Medical University in Harbin, Heilongjiang Province, China. Harbin Medical University provided training in animal experimentation for all researchers who operated with animals. The Animal Experiment Center Ethics Committee of the hospital approved all animal experimentation methods, including the radiation of SD rats at the Radiation Center of Harbin Medical University Cancer Hospital. Initially, the rats were put to sleep with an injection of 5% sodium pentobarbital (0.1 mL/100 g), and the inner thigh's rectus femoris muscle was marked for radiation. The irradiation area was approximately 2 cm × 2 cm, and the radiation dose was 90 Gy. The radiation dose of skin area was selected based on previous studies demonstrating that this dose effectively induces acute radiation dermatitis in SD rats, leading to ulceration, dermal thickening, inflammation, hair follicle loss, and sebaceous gland loss [32, 37]. This model has been widely used for studying radiation-induced skin injury and evaluating potential therapeutic interventions.

36 male SD rats were randomly divided into a negative control (NC) group (normal rats), a Radiation (RT) group, and a RT + ADSC-exos group (n = 12 in each group). Rats in the RT group were injected with 100 mg PBS after 90 Gy irradiation, whereas rats in the RT + ADSC-exos group received with 100 mg ADSC-exos locally 24 h after 90 Gy irradiation. The RT and RT + ADSC-exos groups received a single dose of 90 Gy local irradiation over a 2 × 2 cm skin area. ADSC-exos (100 µg) or PBS (control) was administered as a single local injection 24 h post-irradiation. No additional injections or irradiations were performed.

The degree of damage to the skin tissue after irradiation was independently scored by two individuals who were not informed of the two groups. The skin damage was recorded daily after the operation through photography, and wound scores and images were documented until 4 weeks after the operation. The degree of skin tissue toxicity was scored according to the previously reported skin scoring criteria for animal models (Table 1) [32]. The method of SD rats euthanasia: intraperitoneal injection of three times the anesthetic dose of pentobarbital.

**Table 1:** Score evaluation criteria for radiation‐induced dermatitis of rat skin

Score	Observation
1.0	No effect
1.5	Minimal erythema, mild dry skin
2.0	Moderate erythema, dry skin
2.5	Marked desquamation, minimal dry crusting
3.0	Dry desquamation, minimal dry crusting
3.5	Dry desquamation, dry crusting, superficial minimal scabbing
4.0	Patchy moist desquamation, moderate scabbing
4.5	Confluent moist desquamation, ulcers, large deep scabs
5.0	Open wound, full‐thickness loss
5.5	Necrosis

### Construction of the radiation-sensitive cell model

In the radio-sensitive cell model, it is necessary to determine the optimal irradiation dose and the optimal drug concentration. To determine the optimal irradiation dose and ADSC-exos concentration for human skin fibroblast (HSF) cells, we conducted a preliminary dose–response study using the MTT assay. HSF cells were first exposed to different radiation doses (4 Gy, 6 Gy, and 8 Gy) using a linear accelerator (VARIAN IX, USA). Cell viability was assessed 24 h post-irradiation. The results indicated that at 6 Gy, HSF cells exhibited a significant but not excessive reduction in viability, allowing for the assessment of potential protective effects of ADSC-exos. Higher doses (8 Gy) resulted in excessive cell death, while lower doses (4 Gy) did not induce significant damage. Based on these findings, 6 Gy was selected as the optimal irradiation dose for in vitro experiments. Similarly, to determine the optimal concentration of ADSC-exos, irradiated HSF cells were treated with different concentrations of ADSC-exos (5 μg, 10 μg, and 15 μg). Cell viability was assessed using the MTT assay 24 h post-treatment. The results indicated that 10 μg ADSC-exos provided the most significant protective effect without causing excessive proliferation or cytotoxicity. Therefore, 10 μg was selected as the optimal concentration for subsequent in vitro experiments.

After the successful establishment of the in vitro model, the HSF cells were divided into three groups, namely, NC group, RT group, and RT + ADSC-exos group, and the HSF cells in RT group were spiked with 10 μg of PBS in the culture medium after 24 h of irradiation at 6 Gy, whereas 10 μg of ADSC-exos was spiked in the culture medium after 24 h of irradiation at 6 Gy in RT + ADSC-exos group. For use in in vitro assays employing EDU, PCR, Western blotting (WB), and immunofluorescence, the cells were taken 72 h after radiation.

### Histological staining and immunohistochemical analysis

The excised rat skin tissue was embedded in paraffin, sliced into 4 µm slices, and fixed in 4% paraformaldehyde for a full day. Hematoxylin Eosin staining (HE) and Masson's trichrome (Solarbio, Beijing, China) were used to stain the slices, and an Olympus optical microscope was used to take pictures. The Ki-67 antibody, MMP3 antibody, b-FGF antibody, SDF-1 antibody, HAS1 antibody, HAS2 antibody, and HAS3 antibody (all from Abcam UK) were incubated overnight at 4 °C. The following day, they were exposed to a secondary polyclonal rabbit antibody and photos were taken with a Leica microscope. Image J software was used to perform a quantitative analysis of five randomly chosen locations.

To avoid overcounting the same hair follicle, only tissue sections showing the entire hair follicle from the root to the opening were used for analysis. Sections were selected from comparable anatomical regions across samples. The number of hair follicles per unit area (follicles/mm^2^) was quantified using Image J software on HE stained sections.

Dermal thickness was measured at five randomly selected regions per section using Image J software. Only regions with fully intact dermis were included in the analysis to ensure consistency. Thickness was measured from the epidermal-dermal junction to the upper boundary of the subcutaneous tissue. Sections that did not completely capture the full thickness of the dermis were excluded from analysis.

Collagen fibre content was assessed using Masson's trichrome staining, which selectively stains collagen blue. Five non-overlapping fields per section were randomly selected, and the percentage of collagen-positive area was quantified using Image J software. The average collagen content was reported as the proportion of collagen-stained area relative to the total dermal area.

### Immunofluorescence

A 24-well plate was used to cultivate HSF cells, with 5000 cells per well. They were then treated at room temperature with 4% paraformaldehyde for 10 min, followed by 1% bovine serum albumin (Boster, China) for 30 min. The cells were then incubated with antibodies targeting MMP3, b-FGF, SDF-1, HAS1, HAS2, and HAS3 (purchased from Abcam, UK), followed by goat anti-rabbit immunoglobulin IgG. Thermo Scientific, USA supplied 4',6-diamidino-2-phenylindole (DAPI) for staining the nuclei, and a fluorescence microscope was used to identify brightly colored cells.

### Transmission electron microscopy

Skin tissue was soaked in 2.5% glutaraldehyde phosphate buffer solution and sliced into 50 μm-thick pieces. An electron microscope (Hitachi HT7800/HT7700, Japan) was used to examine the ultrastructure of skin cells and organelles.

### Western-blot

Using RIPA lysis buffer (Beyotime P0013B), proteins were extracted from skin tissue and cells, and the quantities of these proteins were measured using a BCA assay kit (Beyotime China). The proteins were electrophoresed on a sodium dodecyl sulfate–polyacrylamide gel and then placed onto a nitrocellulose membrane. After 5% skim milk was applied to the cell membrane for an hour at room temperature, it was incubated with anti-GAPDH (ab8245, Abcam), anti-β-actin (ab8224, Abcam), anti-CD9 (ab236630, Abcam), anti-CD63 (ab134045, Abcam), anti-TSG101 (ab133586, Abcam), anti-SDF-1 (ab155090, Abcam), anti-MMP3 (ab52915, Abcam), anti-MMP9 (ab76003, Abcam), anti-TGF-β (ab215715, Abcam), anti-IL 10 (ab189392, Abcam), anti-IL 6 (ab214429, Abcam), anti-IL 12 (ab62822, Abcam), anti-PDGF (ab181845, Abcam), anti-bFGF (ab289968, Abcam), anti-VEGF (ab150375, Abcam), anti-SMAD2/3 (ab202445,Abcam), anti-HAS1 (ab198846, Abcam), anti-HAS2 (ab140671,Abcam), anti-HAS3 (ab170872, Abcam). All primary antibodies were selected based on their validated cross-reactivity with rat proteins according to manufacturer datasheets or prior studies.The secondary antibody was goat anti-rabbit IgG (1:8000, Abcam), labeled with peroxidase. The gel was developed and captured on camera using the Bio-Rad USA gel imaging equipment.

### QT-PCR

**Fig. 1:**
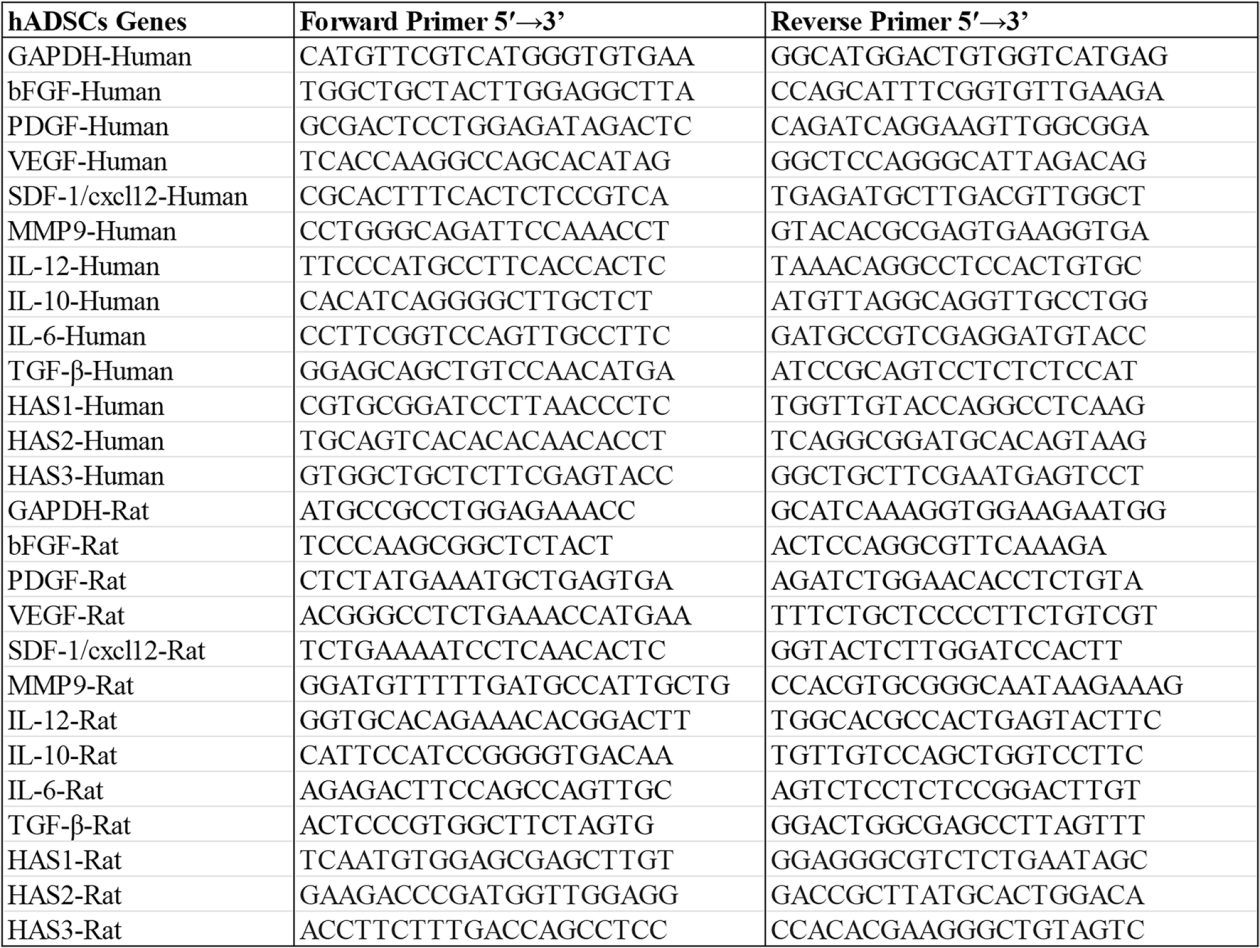
Primer sequences of the study

RNA was extracted from rat skin tissue and HSF utilizing Trizol (TaKaRa, China), followed by cDNA amplification with SYBR Green dye (Roche). The expression levels of SDF-1, MMP3, MMP9, TGF-β, IL-10, IL-6, IL-12, VEGF, PDGF, bFGF, HAS1, HAS2, and HAS3 were systematically assessed using real-time quantitative polymerase chain reaction (qRT-PCR), with primer sequences specified in Fig. 1, alongside the internal controls GAPDH and β-Actin.

### Transwell assay

HSF cells migration was assessed using a 24-well Transwell chamber system with an 8-μm pore polycarbonate membrane insert (Corning, USA). 5,000 cells were seeded in the upper chamber with serum-free medium, while the lower chamber contained medium supplemented with ADSC-exos. After 24 h, the cells on the upper surface of the membrane were carefully removed using a cotton swab, while the migrated cells on the lower surface were fixed with 4% paraformaldehyde, stained with crystal violet, and counted using an optical microscope. Migration was quantified by averaging the number of cells in five randomly selected fields per insert.

### EDU

After exposing the cells to the BeyoClickTM EdU-488 Cell Proliferation Kit, supplement with DAPI solution (1000 ×) and incubate at room temperature in the absence of light for 10 min. Subsequent to cleaning, employ a fluorescence microscope to obtain the photographs.

### Transfection and pathway validation

HSF was dispensed into 6-well plates and incubated overnight in a temperature-regulated cell culture incubator. Upon achieving 70% confluence, the HSF was relocated to a sterile bench, and the supernatant culture medium was discarded. The cells underwent two washes with PBS buffer. The HAS1-targeting siRNA and control siRNA for interference were obtained from Gene Pharma. Cells transfected with Lipofectamine 3000 HSF were plated in six culture dishes and subjected to transfection with either HAS1-targeting siRNA (si-HAS1) or non-targeting control siRNA (si-NC) for durations of 24, 48, or 72 h. The transfection efficiency was assessed by observing the distribution of lipofectamine with a fluorescent microscope. Proteins from the cells were collected and identified after 24 h of culture.

### Statistical analysis

All values are expressed as mean ± SD. We used GraphPad Prism 9.0 software to analyze the data, as well as picture J for statistical analysis and picture processing. To compare the two groups, a paired sample t-test was used. For the three independent control and experimental samples, a one-way analysis of variance was performed. Each experiment was repeated at least three times, with statistical significance indicated by a *P*-value < 0.05.

## Results

After irradiation, all rats survived and their daily activities were normal, including eating and drinking. No instances of paralysis or convulsions were observed in the groups, and there was no significant difference in body weight gain.

### Characterization of ADSC-exos

**Fig. 2:**
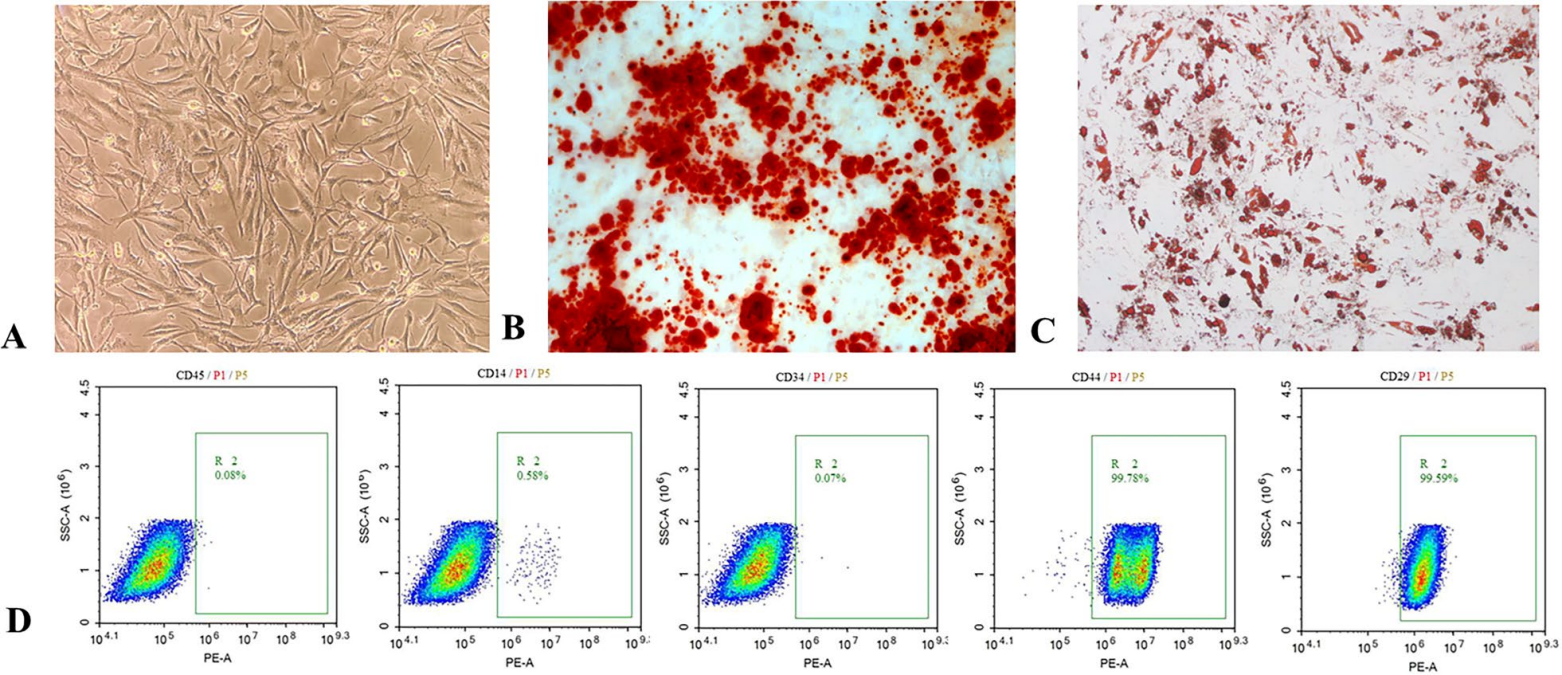
Isolation and characterisation of ADSCs. **A** ADSCs derived from human adipose tissue have a typical spindle form in culture. **B** We studied ADSCs' multi-lineage differentiation potential: alizarin red staining revealed osteogenic differentiation, **C** whereas oil red O staining revealed adipogenic differentiation, Bars, 25 μm. **D** The expression of surface markers on ADSCs was measured using flow cytometry

**Fig. 3:**
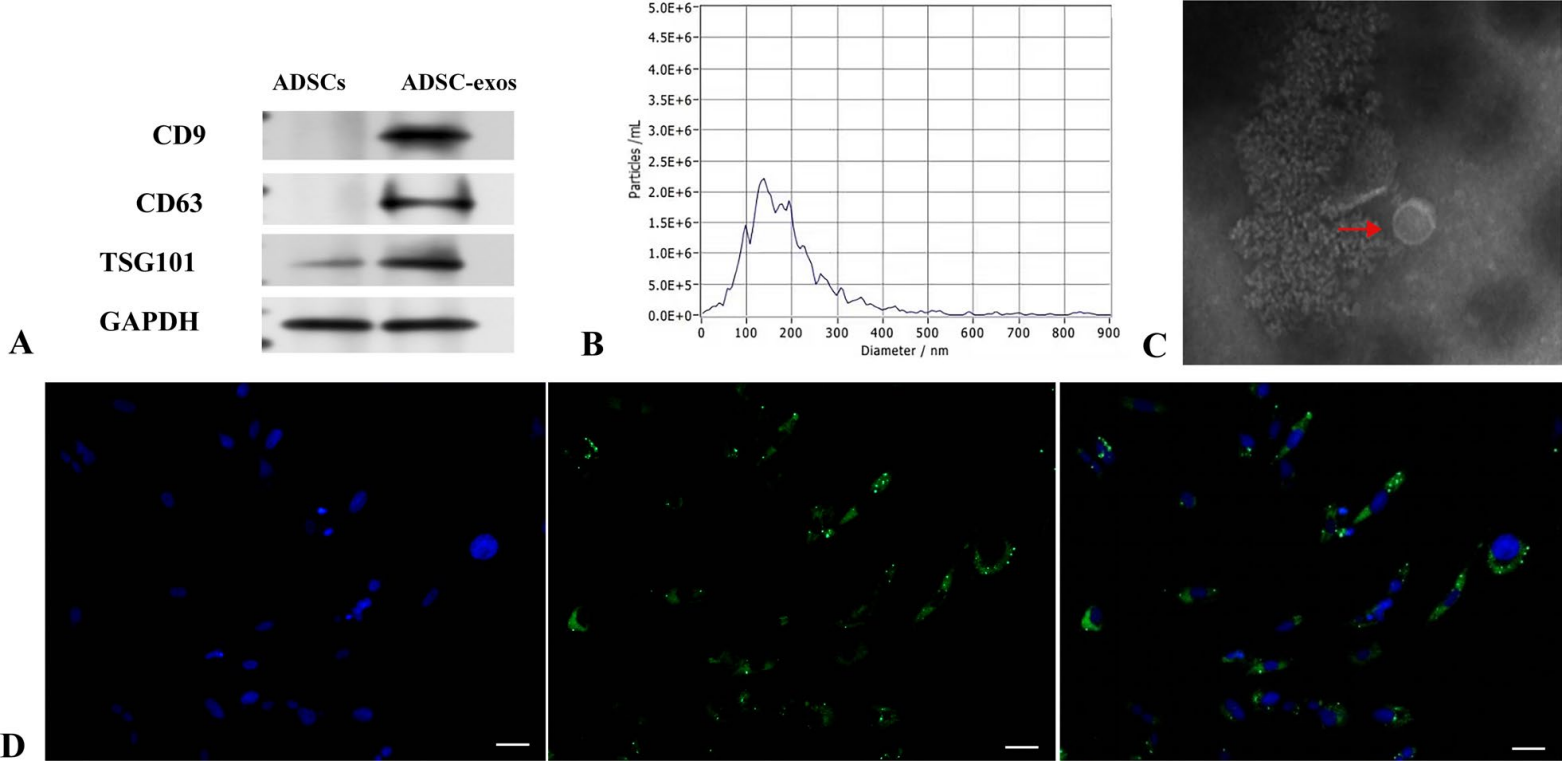
Characterization of ADSC-exos. **A** Western blot analysis of ADSC-exos for the specific markers CD63, TSG101 and CD9. **B** Particle size distribution of ADSC-exos detected by nanoparticle tracking analysis (NTA). **C** Transmission electron microscopy image of ADSC-exos, Bars, 200 μm. **D** PKH67-labeled ADSC-exos were co-cultured with HSFs, and the ADSC-exos were internalized by fibroblasts. Green represents PKH67-labeled ADSCs-exos and blue represents DAPI-labeled HSF nuclei, Bars, 25 μm

ADSCs showed spindle-shaped attachment and growth under the microscope (Fig. 2A). Alizarin red staining indicated that ADSCs formed calcified nodules following 21 days of development, implying osteogenic differentiation (Fig. 2B). Oil Red O staining of ADSCs demonstrated the presence of lipid droplets following 7 days of adipogenic development (Fig. 2C), whereas flow cytometry indicated that ADSCs were positive for CD44, CD29, and CD105, and negative for CD14, CD45, and CD34 (Fig. 2D). Exosomal surface markers TSG101, CD9 and CD63 were detected using immunoblotting, and the results showed that all three markers were highly expressed in purified exosomes (Fig. 3A). Nanoparticle tracking analysis (Nano Sight NS300) was employed to assess the size of the isolated exosomes, indicating an average vesicle diameter between 100 and 170 nm (Fig. 3B). The morphology and size of the exosomes were examined using a transmission electron microscope, revealing cup-shaped or spherical particles (Fig. 3C).The data indicate that the nanoparticles correspond with the specified exosomes. The ADSC-exos were labeled with PKH67 prior to co-culturing with HSF. It was found that ADSC-exos were internalized by fibroblasts, indicating that ADSC-exos can be effectively isolated and transferred to HSF (Fig. 3D).

### ADSC-exos reduces radiation-induced acute skin reactions

**Fig. 4:**
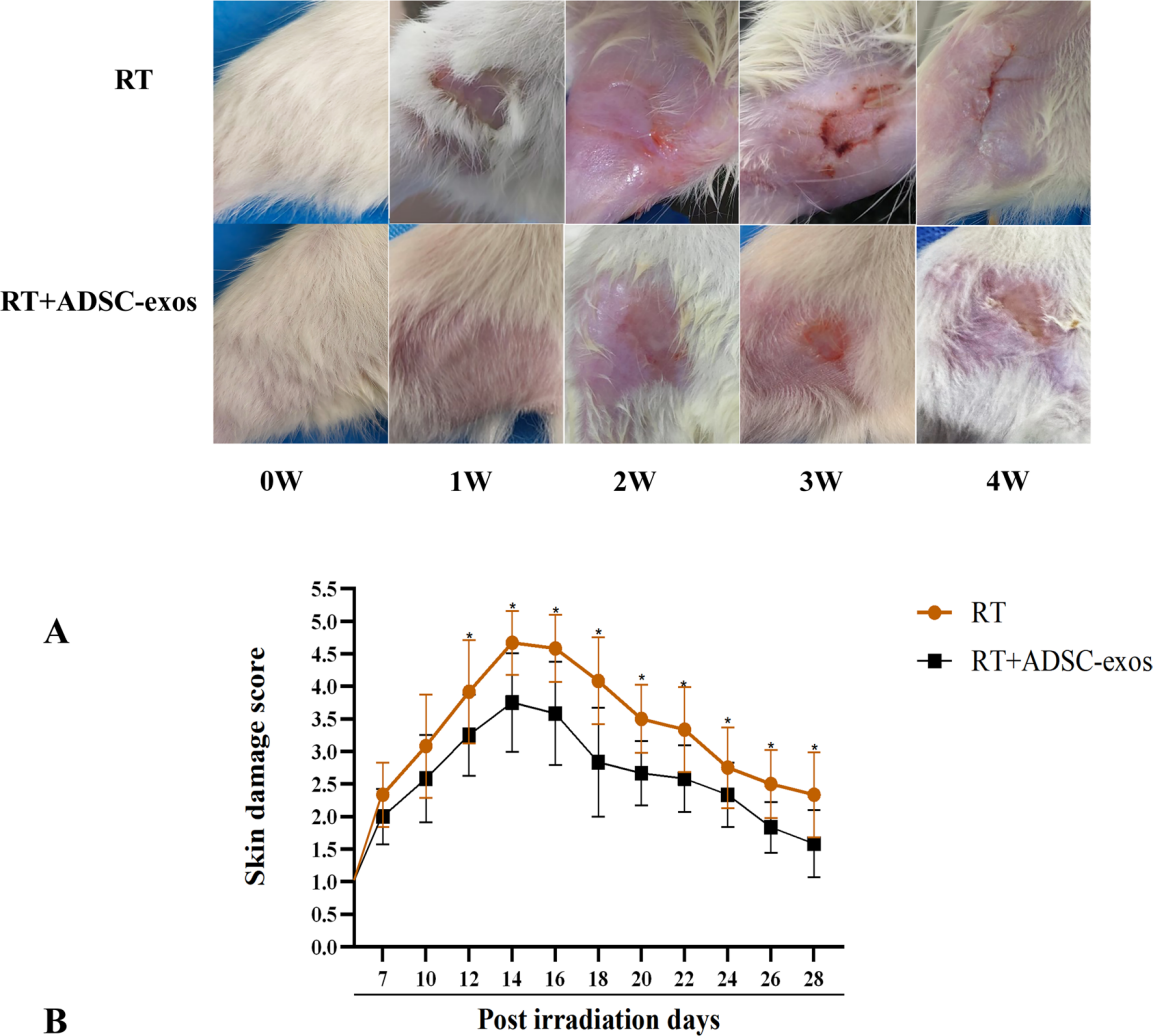
ADSC-exos alleviates skin damage in a rat model of radiation-induced dermatitis. **A** Male rats were irradiated with 90 Gy and either injected with PBS (RT group, n = 12) or injected with 100 mg ADSC-exos (RT + ADSC-exos group, n = 12). Representative images of the skin at weeks 0–4 post-irradiation are shown. **B** The skin injury was semi-quantitatively scored from 1 (no effect) to 5.5 (necrosis). At 4 weeks after irradiation, the skin injury score of the ADSC-exos group was lower than that of the RT group, and the results are expressed as mean ± SD. *p < 0.05, ***p < 0.01, ***p < 0.001 vs. the RT group

**Fig. 5:**
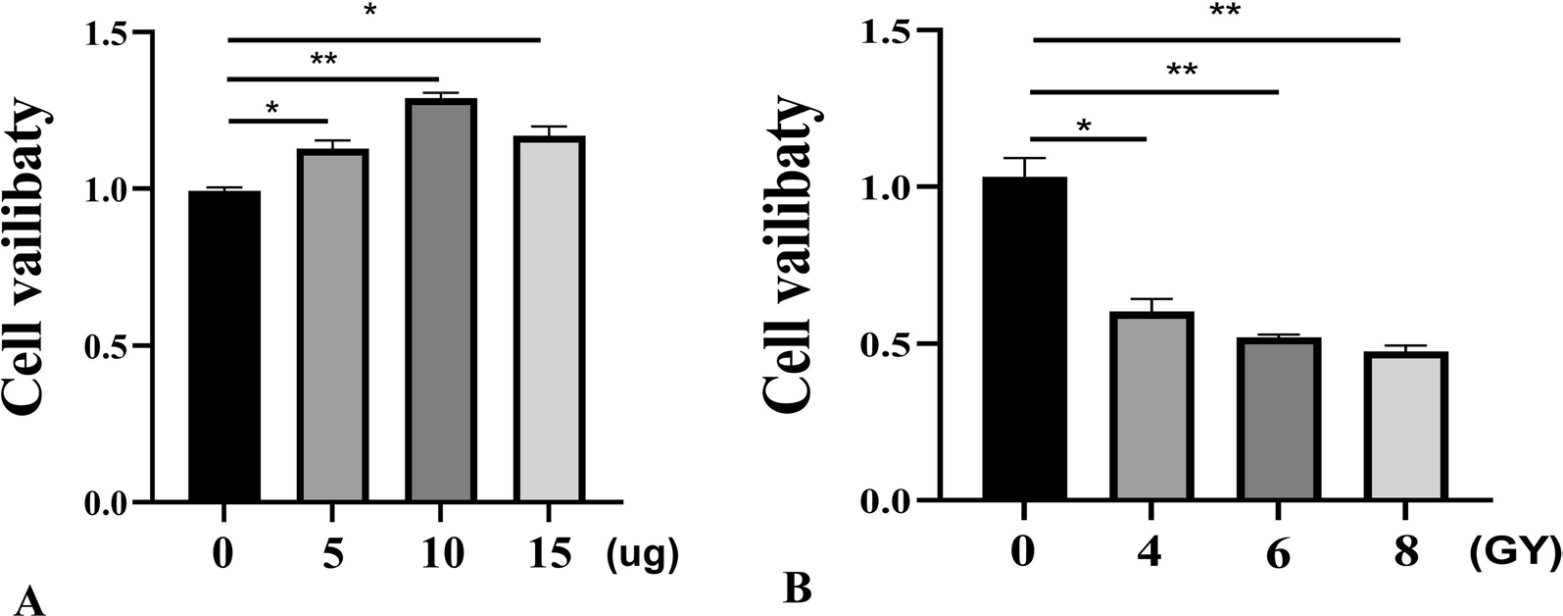
Modeling of radiation in HSF cells. **A** The optimal dosing concentration of ADSC-exos was determined by the MTT assay. **B** MTT assay to determine the optimal irradiation dose of HSF

In the RT group, skin damage was visible 1 week after irradiation, characterized by prominent erythema, desquamation, and alopecia. Two weeks following irradiation, patients reported large ulcers, open sores, and full-thickness skin loss. By the third week following irradiation, skin injury had become less severe. After four weeks of irradiation, scar tissue formed to cure most visible skin damage. In the ADSC-exos group, the skin was mostly normal 1 week after irradiation. At 2 weeks post-irradiation, the skin showed smaller ulcers and open wounds than the RT group. Most visible skin injury was healed by scar tissue within 3–4 weeks of irradiation, earlier than in the RT group (Fig. 4A). The skin damage score during the observation period is shown in Fig. 4B, with the ADSC-exos group showing lower scores compared to the RT group. The optimal concentration of ADSC-exos of HSF cells in the in vitro model was determined using the MTT assay, and the optimal concentration was 10 μg (Fig. 5A). The optimal irradiation dose of HSF cells was 6 Gy (Fig. 5B).

**Fig. 6:**
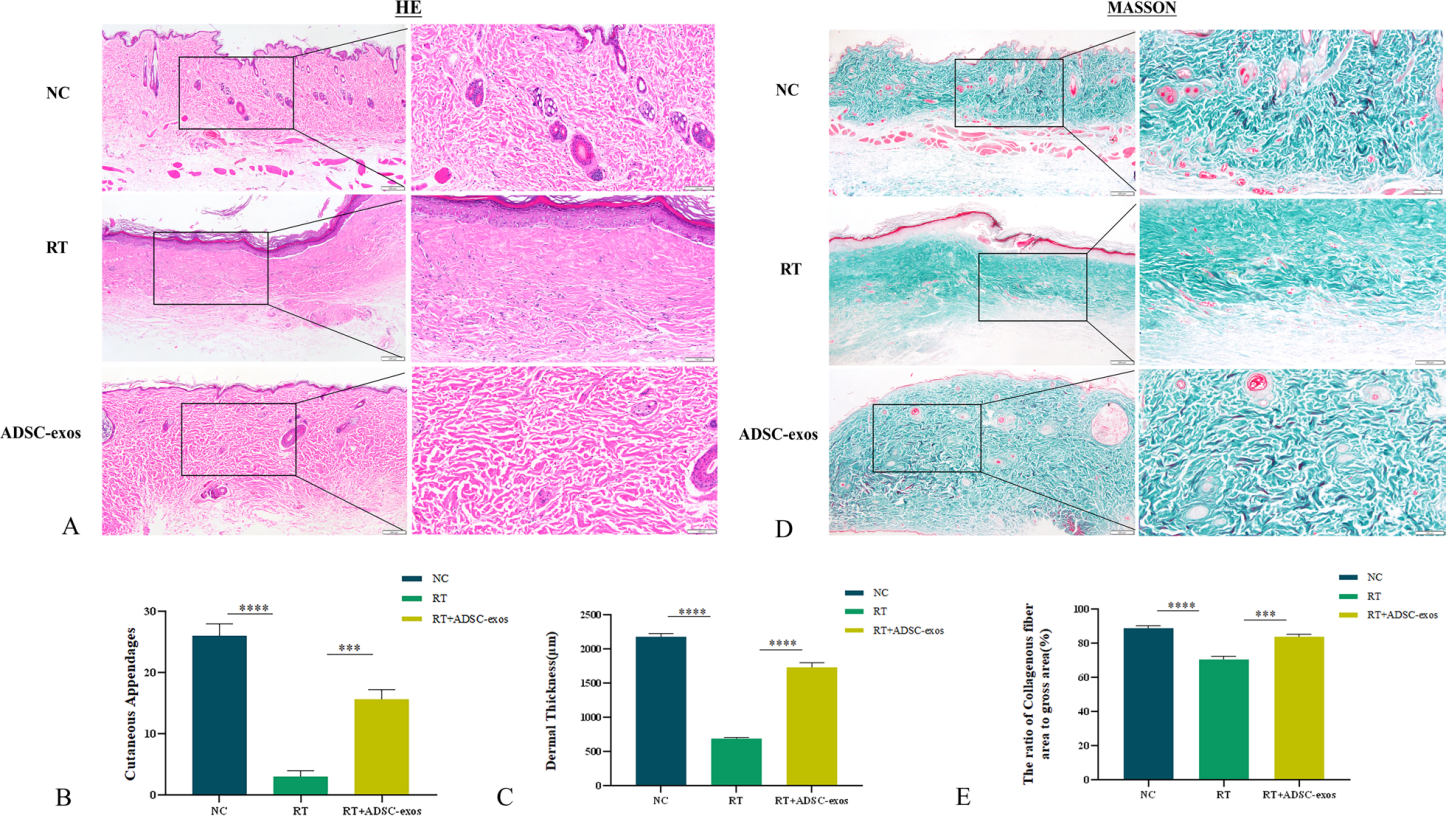
ADSCs reduce radiation-induced dermatitis in vivo. Representative images of hematoxylin and eosin-stained skin from rats four weeks after radiation. **A** Image of HE staining of rat tissue 4 weeks after irradiation (n = 3). **B** Statistical analysis of the number of cutaneous appendages in each group of wound tissue. The results are expressed as mean ± SD. *p < 0.05, ***p < 0.01, ***p < 0.001 vs. the NC group. **C** Statistical analysis of dermal thickness in various wound tissue groups. The results are expressed as mean ± SD. *p < 0.05, ***p < 0.01, ***p < 0.001 vs. the NC group. **D** Masson's trichrome staining of rat tissue 4 weeks after irradiation (n = 3). **E** Statistical analysis of the ratio of the area to the total area of each group of collagen fibres (%). The results are expressed as mean ± SD. *p < 0.05, ***p < 0.01, ***p < 0.001 vs. the NC group

**Fig. 7:**
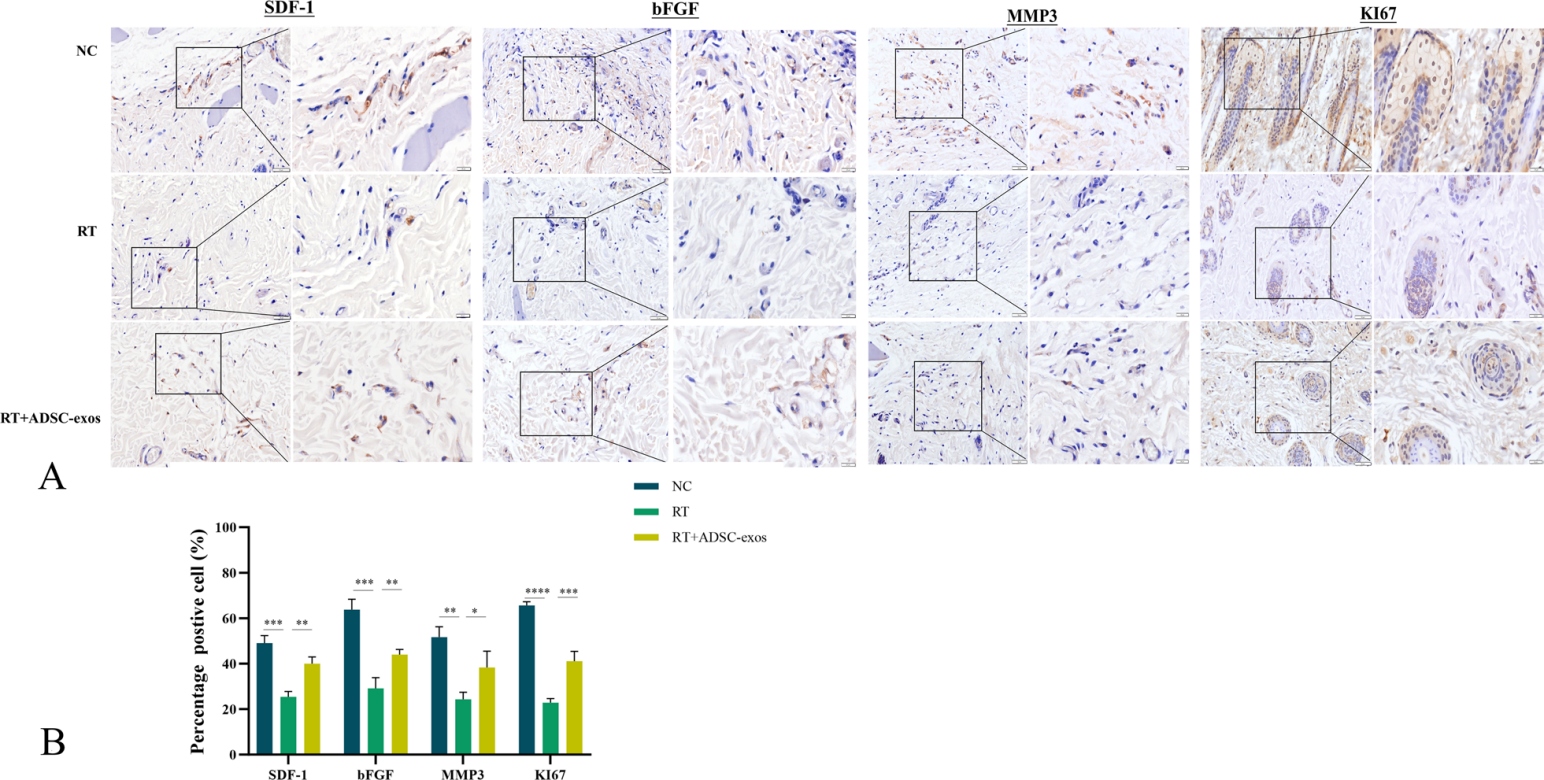
Degree of healing of ARD after irradiation. **A** Representative images of skin tissue immunohistochemically stained for SDF-1, bFGF, MMP3 and Ki67 antibodies (n = 3). **B** In a rat model of radiation-induced dermatitis, RT downregulates the expression of SDF-1, bFGF, MMP3 and Ki67, while ADSC-exos upregulates the expression of SDF-1, bFGF, MMP3 and Ki67. Statistical analysis of positive expression results. For Ki67 staining, the percentage of positive cells was calculated as a total percentage across all skin layers (epidermis, hair follicles, and dermis). The results are expressed as mean ± SD. *p < 0.05, ***p < 0.01, ***p < 0.001 vs. the NC group

**Fig. 8:**
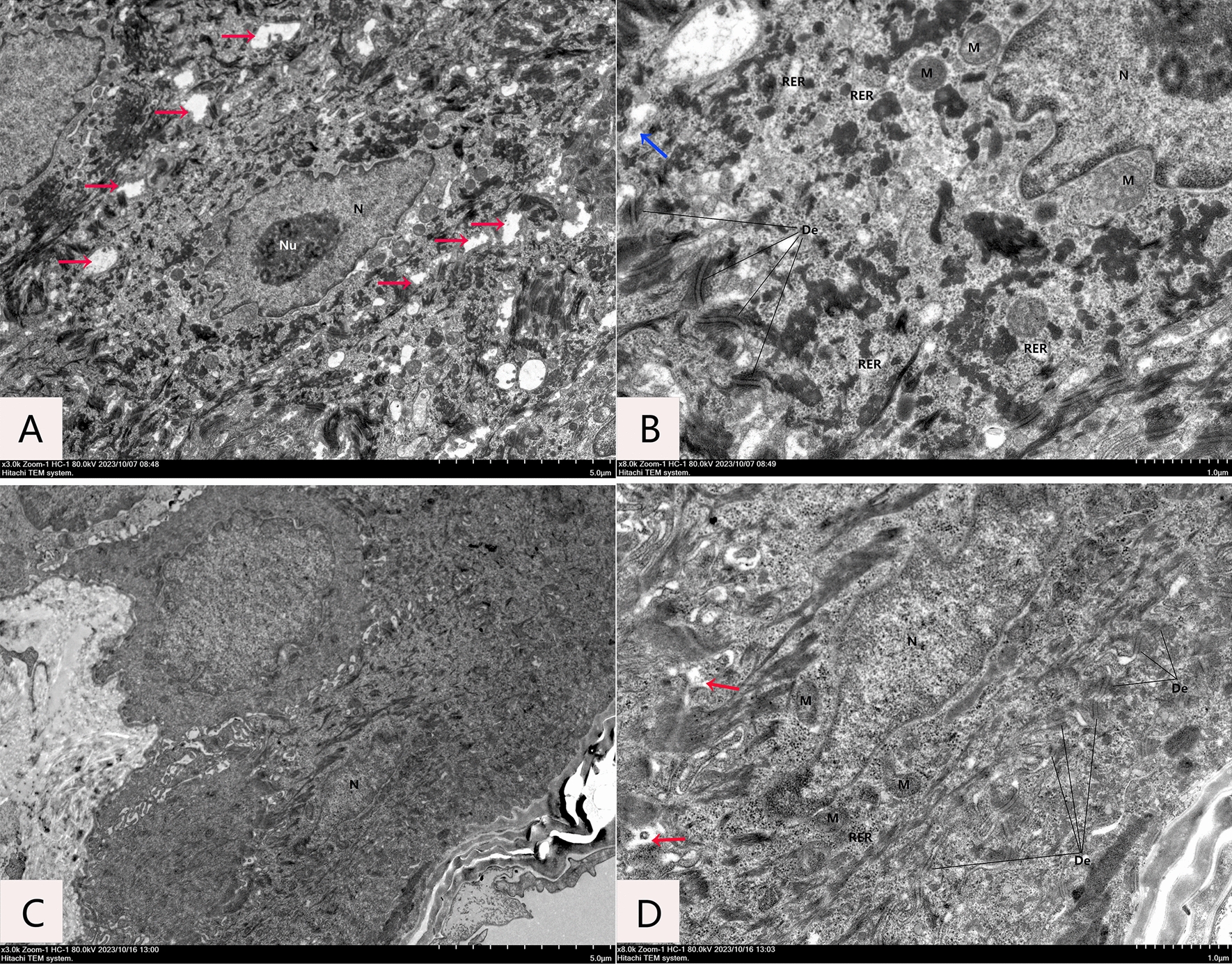
Transmission electron microscopy analysis of the ultrastructure of skin tissue in rats in the RT group and the ADSC-exos group 4 weeks after irradiation (n = 3). The RT group **A**, **B** showed obvious damage to the spiny cells, with local dissolution of the cell membrane (indicated by the blue arrow in (**B**). bridging particles (De) between cells were seen as patches with high electron density, and their number decreased. The intercellular spaces were significantly widened in many places (shown by the red arrow in (**A**). There were fewer intracellular organelles, some of which were obviously swollen, and abundant keratin filaments in the cytoplasm were seen to be degenerated and aggregated. The cell nucleus (N) is irregular in shape, the nuclear perinuclear space is slightly widened, the nucleolus (Nu) is large and central; there are few mitochondria (M), which are severely swollen, with an uneven endoplasmic reticulum matrix, small areas dissolved, and fewer and shorter cristae. The number of rough endoplasmic reticulum (RER) ribosomes is attached. The ADSC-exos group **C**, **D** has less damage to the spiny cells, the cell membranes are intact, and the intercellular bridges (De) are visible as patches of higher electron density distributed symmetrically. The cell spaces are locally widened (indicated by the red arrow in (**D**), and most of the organelles are slightly swollen. The cell nucleus (N) is irregular in shape, the nuclear membrane is intact, and the nuclear perinuclear space is not widened. There are few mitochondria (M), which are slightly swollen, with a uniform matrix. The rough endoplasmic reticulum (RER) is few in number and not significantly expanded

Four weeks after irradiation, skin tissue from NC, RT and ADSC-exos groups was collected for histological analysis (Fig. 6A). The RT group showed epidermal loss, epidermal thickening, lymphocyte infiltration, reduced vascularity, and loss of skin appendages four weeks after irradiation. In contrast, at the same time point, the ADSC-exos group had reduced epidermal loss, less epidermal thickening, less lymphocytic infiltration, more blood vessels, and less loss of skin attachments (Fig. 6B and C). Masson trichrome staining and collagen fiber measurement were performed on rats four weeks after irradiation (Fig. 6D). Figure 5E shows that the ADSC-exos group had a higher percentage of collagen fibers than the RT group. We used immunohistochemical staining to measure levels of Ki67, bFGF, MMP3, and SDF-1 in skin tissue. Our findings showed that 4 weeks post-irradiation, the expression of these markers reduced in the RT group but significantly increased in the ADSC-exos group (Fig. 7). Transmission electron microscopy showed that four weeks after irradiation, the RT group rats' dermal tissue showed damage to spiny cells, including widening of cell spaces (red arrow in Fig. 8A), local membrane dissolution (blue arrow in Fig. 8B), irregular nuclei (N), irregular squamous cells, mitochondrial crest loss, nuclear shrinkage and dissolution, and swelling of the vascular endothelium. In the ADSC-exos group, 4 weeks after irradiation, there was less damage to the spiny cells, the intercellular spaces were partially widened (as indicated by the red arrow in Fig. 8D), there were fewer intracellular organelles, the cell membrane was intact, and the intercellular desmosomes (De) were symmetrically distributed in patches with high electron density. The majority of the organelles exhibited mild swelling (Fig. 8C).

**Fig. 9:**
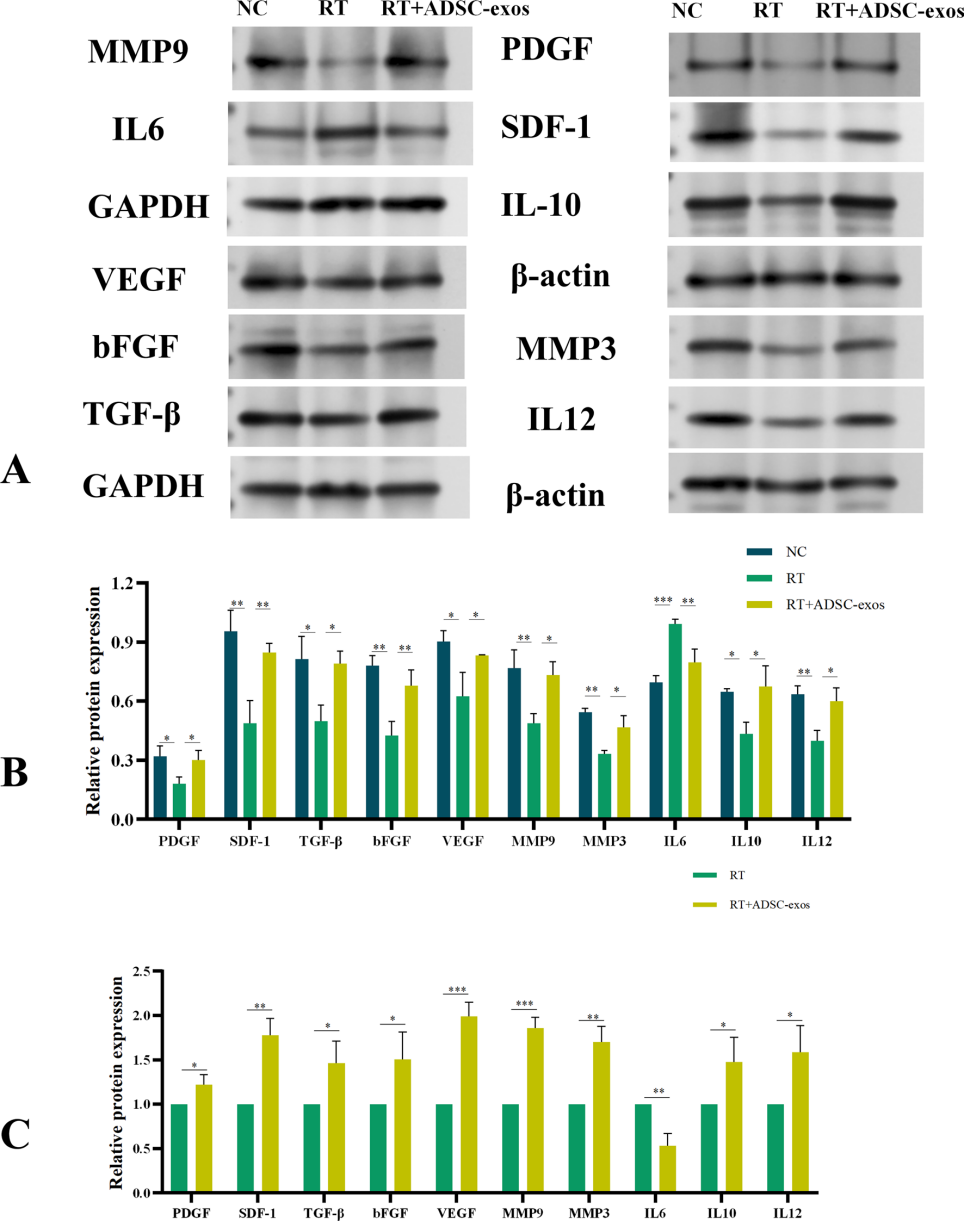
Western Blot results and PCR results of animal model rat tissues. **A** Western-blot analysis of protein levels of bFGF, MMP3, SDF-1, MMP3, MMP9, TGF-β, IL-10, IL-12, VEGF, PDGF levels in skin tissues (n = 3). **B** Statistical analysis of the quantitative protein expression results for each indicator. The results are expressed as mean ± SD. *p < 0.05, ***p < 0.01, ***p < 0.001 vs. the NC group. **C** Statistical analysis of the PCR quantitative expression results of each index. The results are expressed as mean ± SD. *p < 0.05, ***p < 0.01, ***p < 0.001 vs. the NC group

To evaluate the effect of ADSC-exos on wound healing, we used WB to determine the levels of wound-related inflammatory factors, growth factors, and chemokines. Figure 9A and B shows that this group had higher amounts of TGF-β, bFGF, PDGF, MMP3, MMP9, SDF-1, VEGF, and IL-10, but lower levels of IL-6. The data imply that ADSC-exos have a considerable impact on inflammation suppression, promotion of angiogenesis, enhancement of growth factor expression, extracellular matrix remodeling, thus speeding wound healing. RNA was collected from rat tissues in both the RT and ADSC-exos groups. Gene expression of bFGF, SDF-1, MMP3, MMP9, TGF-β, IL-10, IL-12, VEGF, and PDGF was detected by PCR. The results were mostly similar with the WB analysis findings (Fig. 9C).

### ADSC-exos protects HSF cells from radiation induction

**Fig. 10:**
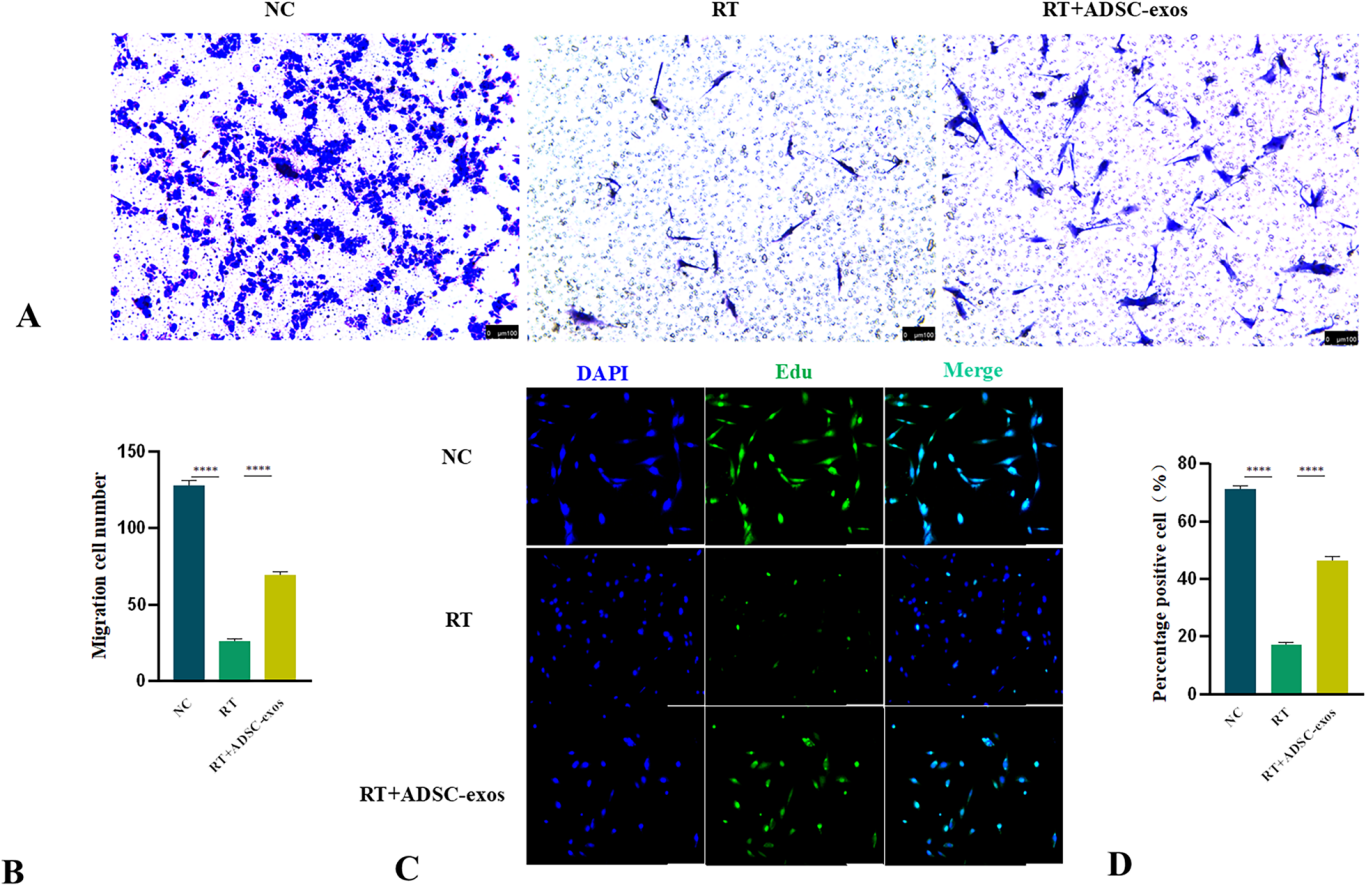
Assessment of the migration and proliferation abilities of HSF cells after treatment. **A** Representative images of transwell (n = 3). **B** The migration ability of cells in the RT group was reduced, and the migration ability of the ADSC-exos group was enhanced compared to the RT group. The results are expressed as mean ± SD. *p < 0.05, ***p < 0.01, ***p < 0.001 vs. the NC group. **C** Representative images of the EDU experiment under immunofluorescence (n = 3). **D** HSF cells have reduced proliferative capacity after irradiation, which is enhanced after the addition of ADSC-exos. The results are expressed as mean ± SD. *p < 0.05, ***p < 0.01, ***p < 0.001 vs. the NC group

**Fig. 11:**
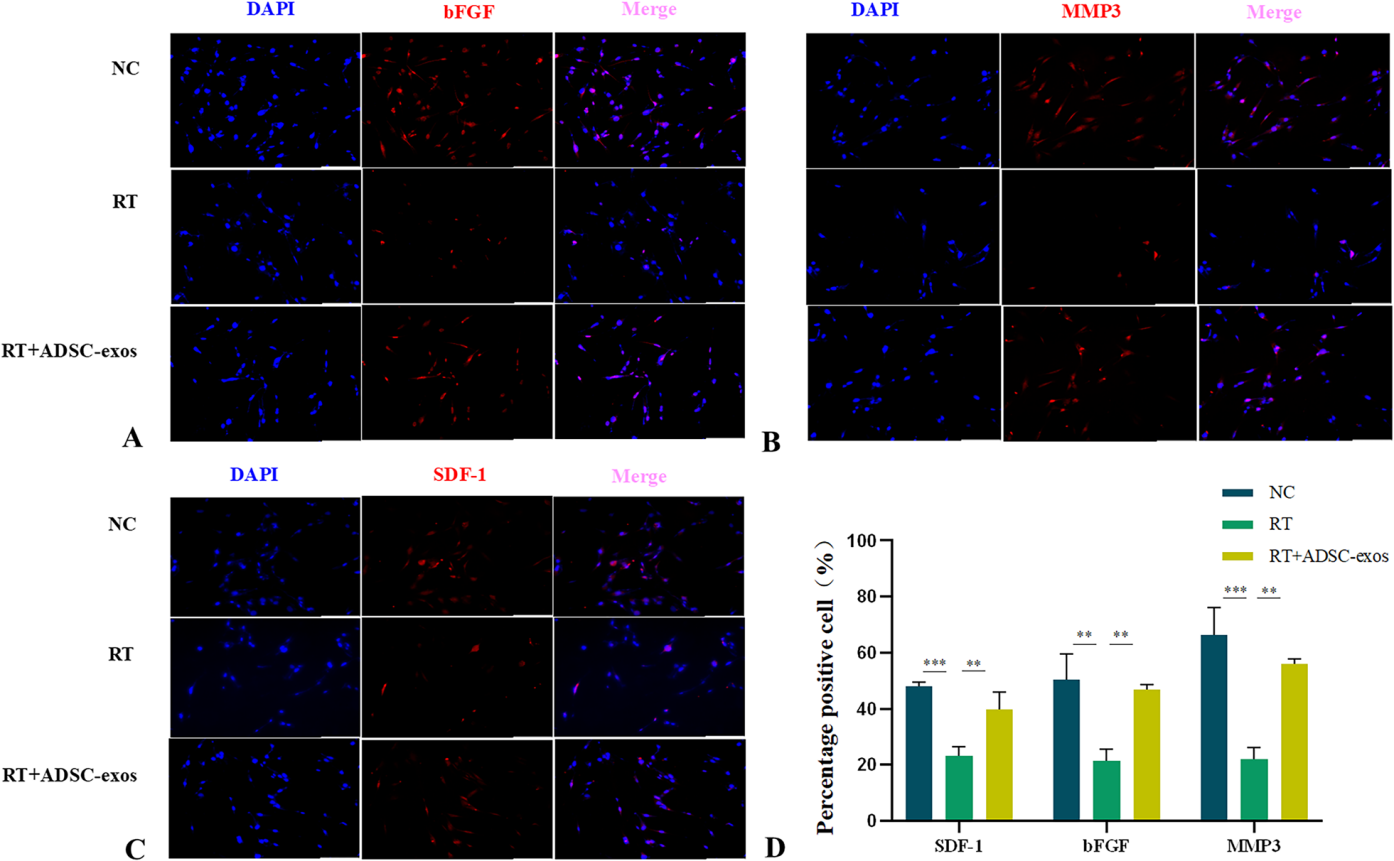
Immunofluorescence results. **A**, **B**, **C** Representative images of immunofluorescence staining of SDF-1, bFGF, and MMP3 (n = 3). **D** In the radiation-induced HSF cell model, RT downregulates the expression of SDF-1, bFGF, and MMP3 in cells, while ADSC-exos upregulates the expression of SDF-1, bFGF, and MMP3. The results are expressed as mean ± SD. *p < 0.05, ***p < 0.01, ***p < 0.001 vs. the NC group

Initially, we established a radiation-induced HSF cell model. Thereafter, the irradiated cells were administered ADSC-exos. Cellular motility and proliferation were assessed 72 h post-radiation treatment. Transwell was used to detect the migration ability of HSF cells, revealing that migration ability decreased after irradiation, and increased after the addition of exosomes (Fig. 10A and B). The EDU experiment was conducted to assess proliferation capacity of HSF cells after irradiation, and the proliferation ability of the ADSC-exos group increased compared with the RT group (Fig. 10C and D). The expression levels of SDF-1, bFGF, and MMP3 in the NC group, RT group, and ADSC-exos group were evaluated by immunofluorescence. Four weeks after irradiation, the expression of the three markers was lower in the RT group than in the NC group, but it rose dramatically with the addition of ADSC-exos (Fig. 11).

**Fig. 12:**
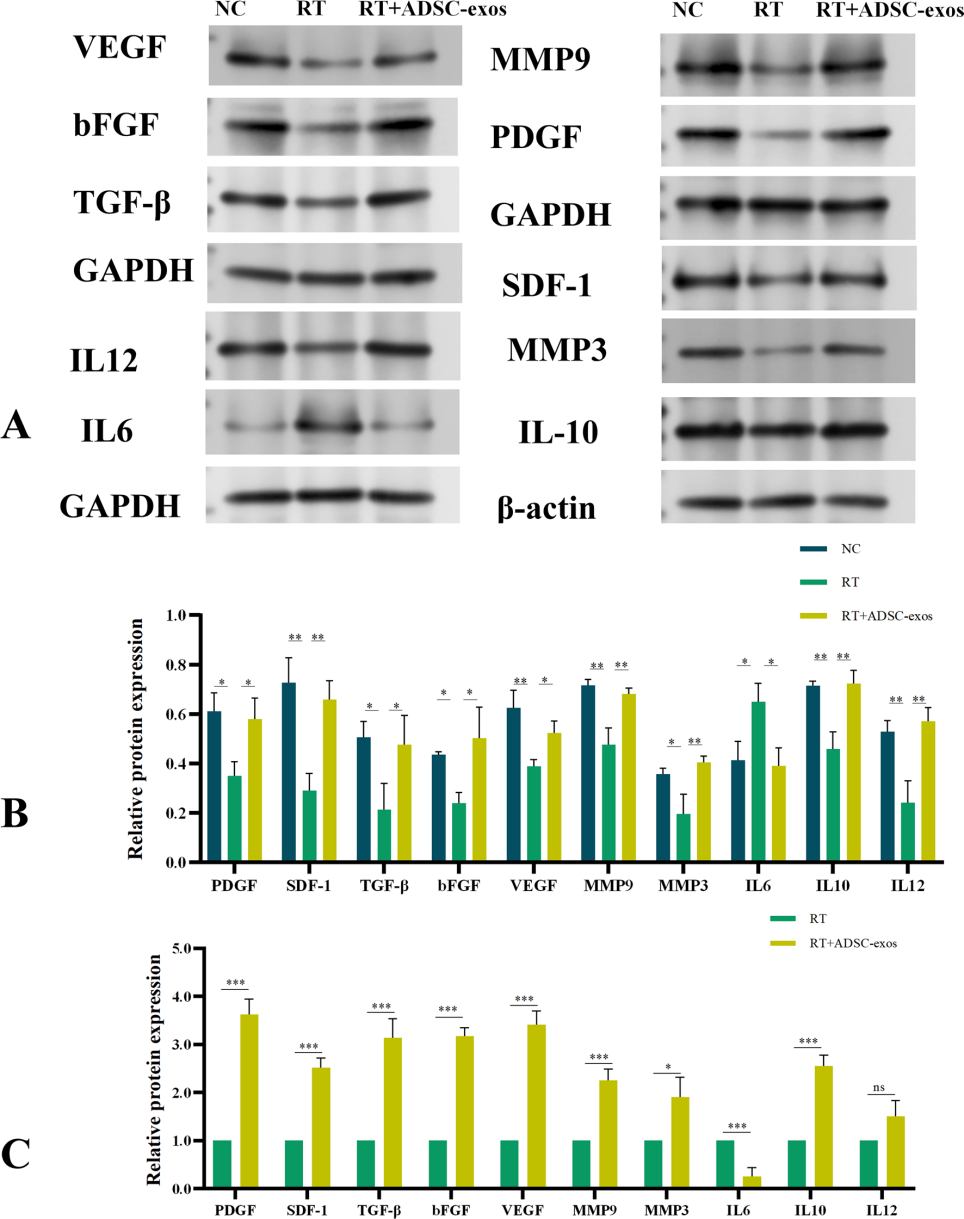
Western Blot results and PCR results of HSF cells. **A** Western-blot analysis of protein levels of bFGF, MMP3, SDF-1, MMP3, MMP9, TGF-β, IL-10, IL-12, VEGF, PDGF levels in HSF cells (n = 3). **B** Statistical analysis of the quantitative protein expression results for each indicator. The results are expressed as mean ± SD. *p < 0.05, ***p < 0.01, ***p < 0.001 vs. the NC group. **C** Statistical analysis of the PCR quantitative expression results of each index. The results are expressed as mean ± SD. *p < 0.05, ***p < 0.01, ***p < 0.001 vs. the NC group

Proteins were isolated from HSF cells in each group four weeks post-irradiation for Western blot tests. The expression levels of bFGF, MMP3, MMP9, SDF-1, TGF-β, IL-10, IL-12, VEGF, and PDGF in the ADSC-exos group were considerably elevated compared to the RT group. The expression of IL-6 in the ADSC-exos group was markedly lower than in the RT group (Fig. 12A and B). Simultaneously, RNA was extracted from HSF cells in each group four weeks post-irradiation, reverse transcribed, and subsequently subjected to PCR to assess the expression of genes including bFGF, MMP3, MMP9, SDF-1, TGF-β, IL-10, IL-12, VEGF, and PDGF, yielding results that were largely congruent with those obtained from WB (Fig. 12C).

### ADSC-exos promotes HAS1 expression to attenuate radioactive skin damage

**Fig. 13:**
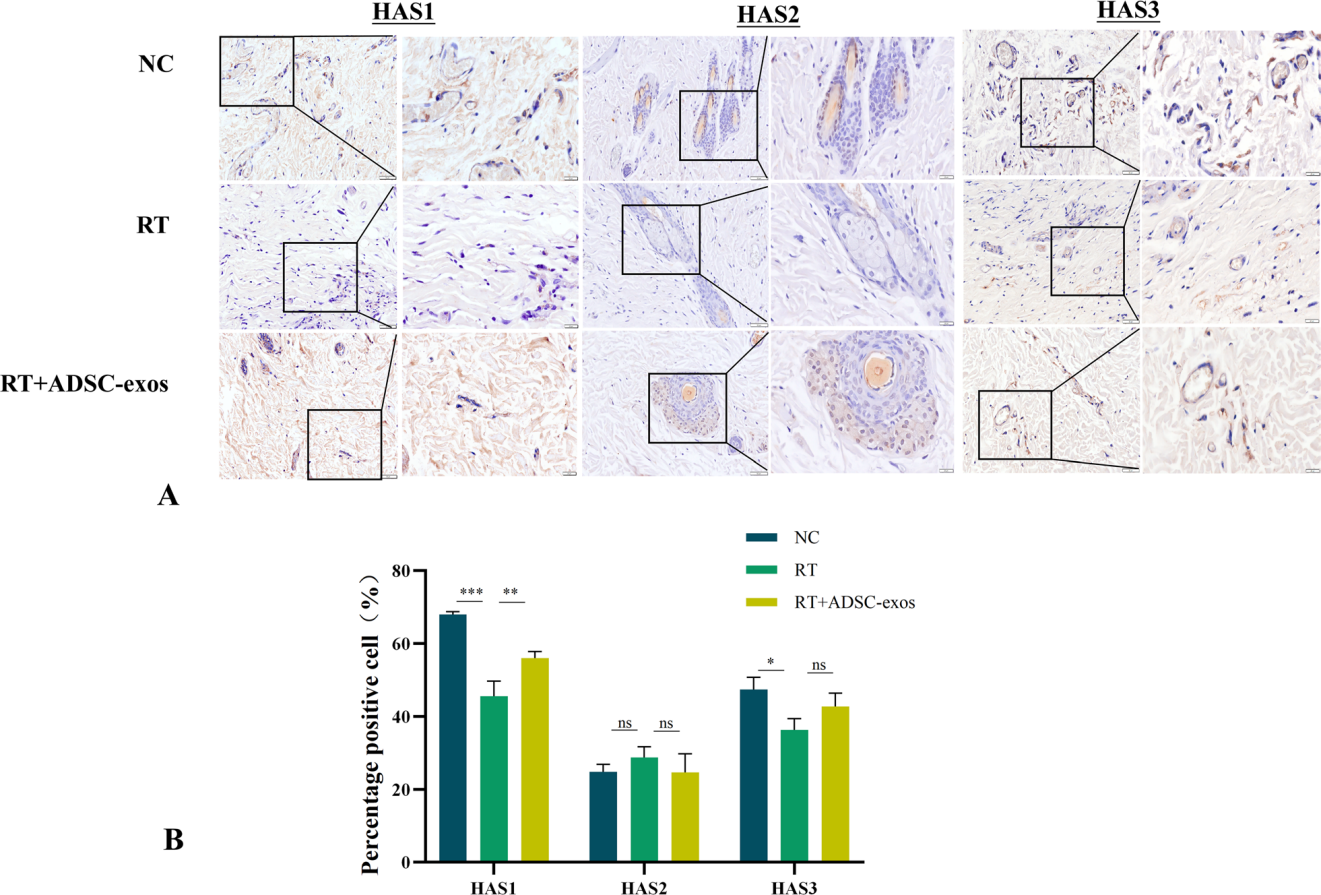
Immunohistochemical detection of HSA expression in tissues from each group of rats. **A** Representative images of skin tissue immunohistochemically stained for HAS1, HAS2 and HAS3 antibodies (n = 3). **B** In a rat model of radiation-induced dermatitis, HAS1 expression was reduced in the RT group and increased in the ADSC-exos group. The expression of HAS2 and HAS3 was not statistically significant. The results are expressed as mean ± SD. *p < 0.05, ***p < 0.01, ***p < 0.001 vs. the NC group

**Fig. 14:**
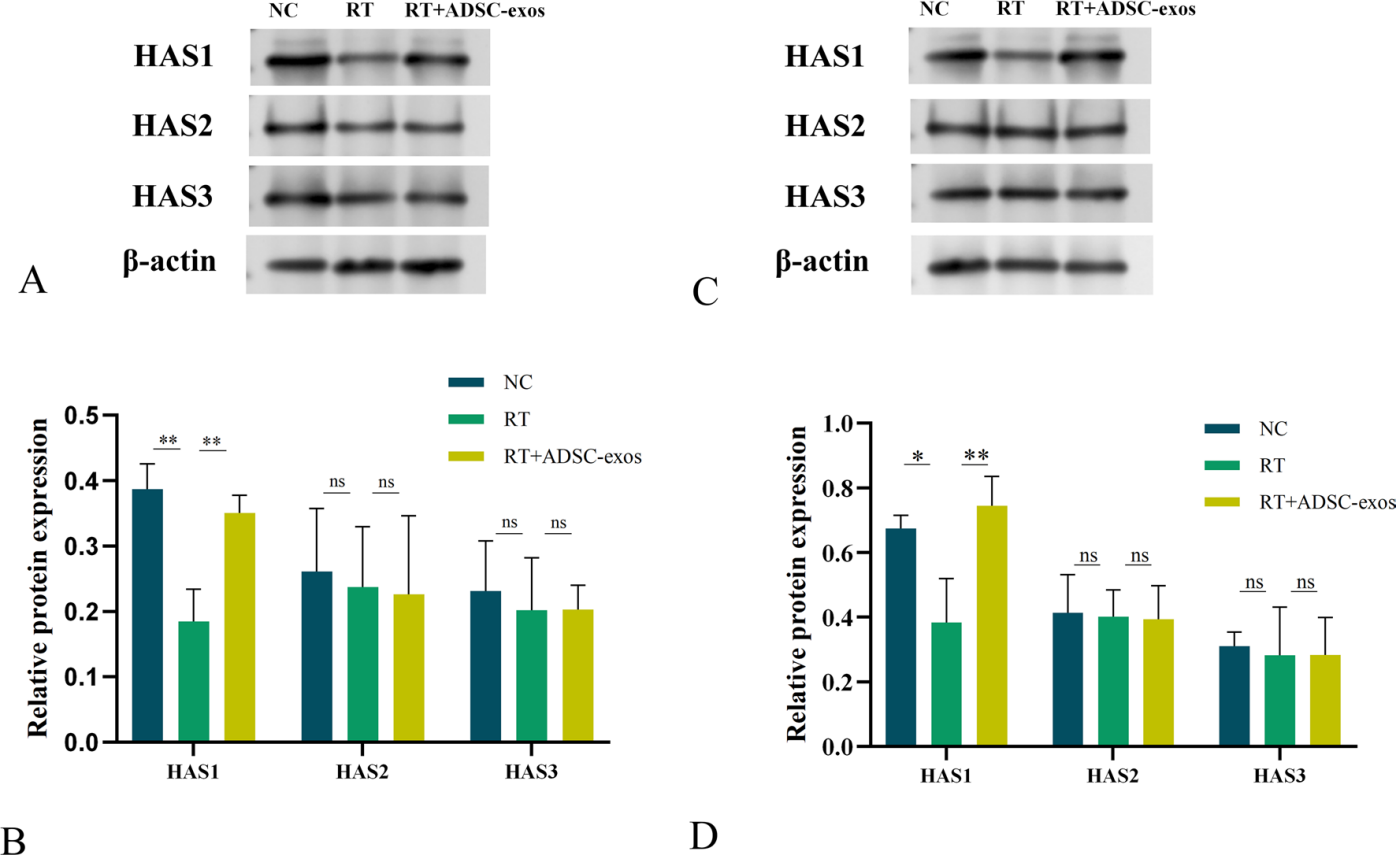
Western blot results for tissues and cells. **A** Representative blots of HAS1, HAS2, and HAS3 levels in the tissue (n = 3). **B** HAS1 expression decreased in the RT group but rose in the ADSC-exos group. The expression of HAS2 and HAS3 did not show any statistical significance. Results are presented as mean ± SD. *p < 0.05; ***p < 0.01, ***p < 0.001 vs. the NC group. **C** Representative blots of HAS1, HAS2, and HAS3 levels in HSF cells (n = 3). **D** HAS1 expression decreased in the RT group but rose in the ADSC-exos group. The expression of HAS2 and HAS3 did not show any statistical significance.Results are presented as mean ± SD. *p < 0.05, ***p < 0.01, and ***p < 0.001 vs. the NC group

**Fig. 15:**
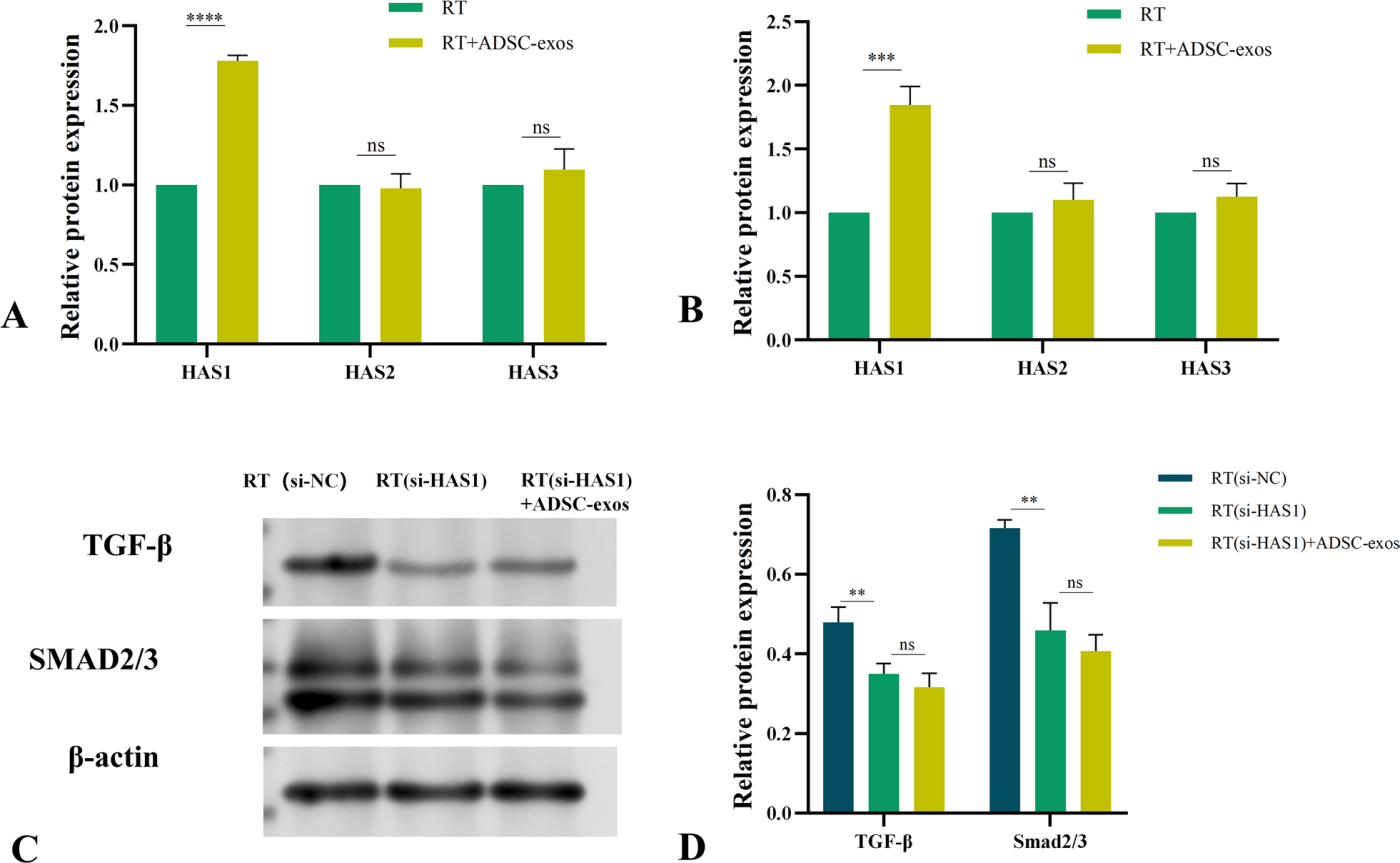
Expression of PCR results of tissues and cells, and verification of related pathways. **A** In the rat tissue, HAS1 PCR expression was increased in the ADSC-exos group. The expression of HAS2 and HAS3 was not statistically significant. The results are expressed as mean ± SD. *p < 0.05, ***p < 0.01, ***p < 0.001 vs. the RT group. **B** In the HSF cells, HAS1 PCR expression was increased in the ADSC-exos group. The expression of HAS2 and HAS3 was not statistically significant. The results are expressed as mean ± SD. *p < 0.05, ***p < 0.01, ***p < 0.001 vs. the RT group. **C** Representative blots ofTGF-β and Smad2/3 levels in the si- HSF cells (n = 3). **D** TGF-β and Smad2/3 expression was reduced in RT (si-HAS1), and the addition of ADSC-exos did not increase expression. The results are expressed as mean ± SD. *p < 0.05, ***p < 0.01, ***p < 0.001 vs. the RT group

HAS1, HAS2, and HAS3 are the members of the hyaluronic acid synthase family. To determine which synthase is most affected by ADSC-exos, we used WB, PCR, and immunohistochemical staining to detect the expression of HAS1, HAS2, and HAS3. Among them, the immunohistochemical experiments on rat tissues found that the expression of HAS1 and HAS3 decreased after irradiation and increased after the addition of exosomes, but the change in HAS1 expression was more pronounced, while the results for HAS3 were not statistically significant (Fig. 13). The ADSC-exos group exhibited the most substantially upregulated marker, HAS1, as evidenced by the Western blot experiments on rat tissue and HSF cells (Fig. 14). Further, the Western blot findings were consistent with the PCR results (Fig. 15A and B) from in vivo and in vitro experiments. The results presented above suggest that HAS1 is the primary mechanism by which exosomes exert their effects. We selected the classic TGF-β/SMAD2/3 pathway for verification in order to illustrate that ADSC-exos specifically target HAS1. To further explore the role of HAS1 in the regulatory network of ADSC-exos, we performed siRNA-mediated HAS1 knockdown in HSF cells. Western blot analysis showed that siRNA-induced inhibition of HAS1 led to a significant reduction in TGF-β expression and Smad2/3 phosphorylation levels (Fig. 15C and D). These results indicate that HAS1 is not merely a downstream effector but plays an active role in maintaining the activation of the TGF-β/Smad2/3 pathway. This suggests a possible positive feedback loop, where HAS1 expression further enhances TGF-β/Smad2/3 signaling, thereby promoting extracellular matrix remodeling and wound healing.

## Discussion

The appearance of acute radiation skin damage poses a dual challenge to cancer patients and represents an urgent clinical issue in need of resolution. CRD often comes with severe skin fibrosis, for which drug treatment is often ineffective. Moreover, prolonged ulcers can increase the risk of skin malignancies. Therefore, we believe that preventing and alleviating radiation skin damage holds significant clinical value. Both BMSCs and ADSCs are regarded as superior stem cell sources for regenerative medicine and tissue engineering [33]. However, BMSCs have the disadvantages of low stem cell yield and limited available tissue. ADSCs are a better stem cell source due to their advantages of greater tissue availability, higher stem cell concentration, and reduced morbidity at the donor site. Multiple research studies have examined the utilization of ADSCs for the remediation of radiation-induced dermal injury. Sultan et al. shown that fat grafting can alleviate both acute and chronic radiation dermatitis in a murine model [34]. Human-derived mesenchymal stem cells promote healing in the early stages of radiation-induced skin condition [35]. Huang et al. discovered that ADSCs expedite the healing of radiation-induced ulcers by enhancing angiogenesis [36]. ADSCs reduce tissue protease factor F levels and prevent cell death, which helps to radiation dermatitis [37]. Adipose mesenchymal stem cell-conditioned media enhances the healing of radiation-induced skin lesions in rats [11].

ADSCs' ability to regenerate damaged tissue is mostly attributed to their secretome's autocrine and paracrine actions, with ADSC-exos playing an essential part. Exosomes derived from human ADSCs have been demonstrated to stimulate skin fibroblast migration and proliferation [38]. By triggering the SIRT1 pathway, ADSC-exos reduce radiation-induced brain damage [39]. One of the key mechanisms through which ADSCs exert their therapeutic effects is the secretion of growth factors and cytokines, which contribute significantly to wound healing and tissue regeneration. Growth factors such as TGF-β, bFGF, VEGF, and PDGF play critical roles in promoting angiogenesis, extracellular matrix remodeling, and fibroblast proliferation. Additionally, ADSCs secrete anti-inflammatory cytokines like IL-10 and IL-12, which help modulate the immune response and reduce inflammation, thereby promoting an optimal wound-healing environment. These factors collectively enhance tissue repair and improve the regeneration of damaged skin following radiation exposure.

But limited studies have investigated on the direct treatment of radiation dermatitis with ADSC-exos and the related mechanisms of action. In this study, we used ADSC-exos for the first time to treat a rat model of radiation and a radiation cell model. ADSC-exos effectively reduce the severity of ARD in rats by promoting extracellular matrix repair, angiogenesis, hair follicle and sebaceous gland regeneration, inflammation inhibition, and reducing radiation damage to HSF cells. It also improves the ability of HSF cells to grow and migrate.All of these findings point to the fact that ADSC-exos plays a significant role in ARD mitigation. Our study demonstrated that ADSC-exos significantly upregulate the secretion of bFGF, SDF-1, and MMP3 in irradiated HSF cells, which play essential roles in wound healing through fibroblast proliferation, angiogenesis, and extracellular matrix remodeling. Furthermore, immunohistochemical analysis in rat tissue confirmed that the expression patterns of these factors were consistent with the immunofluorescence results, indicating that ADSC-exos regulate these regenerative factors at both cellular and tissue levels. The sustained expression of MMP3 and MMP9 at 4 weeks suggests that ECM remodeling is still active, supporting angiogenesis and final tissue organization. Additionally, IL-12 remains elevated, likely reflecting its role in immune regulation and infection prevention, ensuring complete resolution of inflammation. These findings indicate that while ADSC-exos promotes faster healing, tissue remodeling continues beyond the observed period.

ECM accumulation and circulation are common features of tissue damage, healing, and remodeling in several human disorders. HA is a major component of ECM and plays a critical role in regulating tissue injury, facilitating repair, and modulating disease outcomes. HA's function depends on its molecular size, geographical position, and interactions with binding partners. Cell-surface HA shields tissues from environmental damage while promoting regeneration and repair. The interaction of HA and its related proteins contributes to the development of a wide range of human illnesses [40]. Although the primary structure of HA is simple, it regulates a wide range of biological responses in a complex and nuanced manner. Extracellular HA plays a pivotal role in regulating cell–cell and cell–matrix interactions, controlling migration, proliferation, apoptosis, epithelial-to-mesenchymal transition and stem cell function [41].

In mammals, three multi-spanning transmembrane enzymes (HAS1, HAS2, and HAS3) synthesize it. HAS3 activity is necessary for the production of small molecular weight HA, which can impact the inflammatory response during ventilator-induced lung damage [42]. After destroying the HAS2 gene in stromal fibroblasts, some scholars found that there was a significant impairment in macrophage recruitment, which indicates the contribution of interstitial-derived HA in the mobilization of macrophages in tumors [43]. Mouse epidermal keratinocytes exhibit significant expression of HAS3 mRNA throughout the basal cell layer and extending to the granular cell layer. The results demonstrate that HAS3 gene expression is essential for regulating hyaluronic acid production in the epidermis [44]. Some scholars have found that HAS1 expression is absent in mucosal fibroblasts, but is detected in dermal fibroblasts [45]. Both HAS1 and HAS2 mRNA are detected in the dermis and epidermis of murine skin. When cultured with TGF-β stimulation, the mRNA of HAS1 and HAS2 in fibroblasts is upregulated, though their expression patterns differ, indicating that the HAS1 and HAS2 genes are independently regulated, and the synthesized hyaluronic acid also has different functions in the epidermis and dermis [46]. Research suggests that HAS1 may have a role in inflammation by interacting with cytokines and other variables [47]. To demonstrate the effect of ADSC-exos on the hyaluronan synthase family HAS1, HAS2, and HAS3, we performed immunohistochemistry, WB, PCR, and other experiments, and ultimately found that it exerted the most pronounced effect on HAS1. Our findings indicate that ADSC-exos significantly upregulates HAS1, which is associated with the activation of TGF-β/Smad2/3 signaling. Interestingly, when HAS1 was silenced (si-HAS1 group), the activation of TGF-β and phosphorylation of Smad2/3 were significantly reduced, suggesting that HAS1 is necessary for ADSC-exos-mediated TGF-β/Smad2/3 signaling. Furthermore, our results suggest that in the context of ADSC-exos treatment, HAS1 acts upstream of TGF-β/Smad2/3, possibly initiating a positive feedback loop that enhances tissue regeneration. Further studies are required to elucidate whether TGF-β activation is solely dependent on HAS1 or if additional pathways are involved.

However, there are some limitations in this study. For instance, this study utilized exosomes derived from human adipose-derived stem cell lines. Individual factors of patients, such as advanced age, obesity, and disease, may negatively impact the regenerative potential of ADSCs in terms of growth and differentiation. Here, we demonstrated that ADSC-exos have a protective effect on ARD by promoting angiogenesis and mitigating inflammation. Furthermore, ADSCs can upregulate HAS1 expression to regulate ECM homeostasis and protect the skin. However, the specific growth factors or microRNAs secreted by ADSC-exos that directly target HAS1 have yet to be elucidated. Therefore, in the future, we aim to further investigate the specific mechanism of action of HAS1 on radiation dermatitis and its role in other related diseases.

## Conclusion

This study shows that ADSC-exos can prevent and alleviate the occurrence of ARD by promoting related vascular factors, growth factors, inhibiting inflammation, and regulating extracellular matrix remodeling, and that ADSC-exos can protect against radiation-induced skin damage by upregulating HAS1 through the TGF-β/SMAD2/3 pathway. In conclusion, ADSC-exos is a promising cell-free therapeutic technique for avoiding ARD. This study offers a fresh and complementary perspective for basic research on ARD treatment.

## Supplementary Information

Supplementary Material

## Data Availability

The datasets during and/or analysed during the current study available from the corresponding author on reasonable request.
