# Supplementary figures and images for: Exosomes derived from adipose-derived stem cells alleviate acute radiation-induced dermatitis through up-regulating hyaluronic acid synthase 1 expression
Source: Stem Cell Res Ther. 2025 May 20;16:253. doi: 10.1186/s13287-025-04276-8 (PMC12093883; doi:10.1186/s13287-025-04276-8)

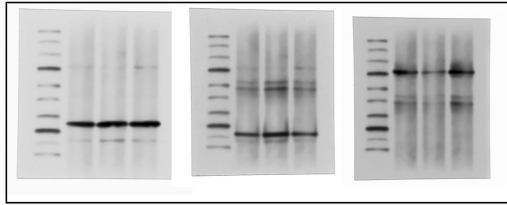

**A**

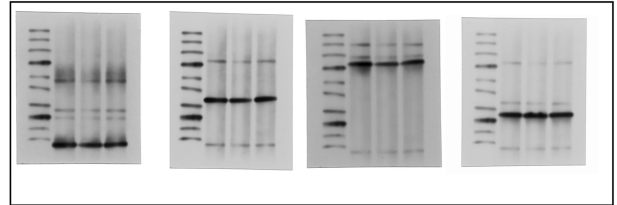

**B**

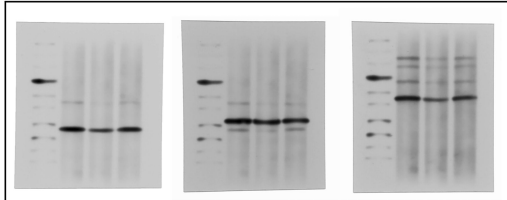

**C**

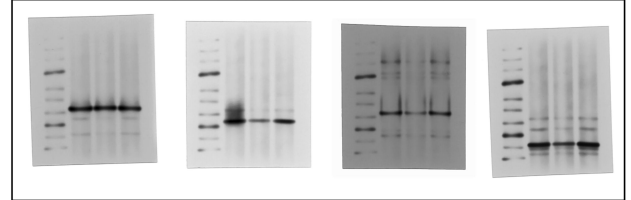

**D**

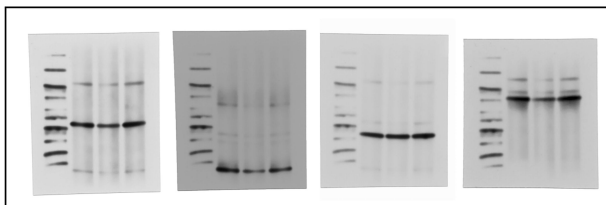

**E**

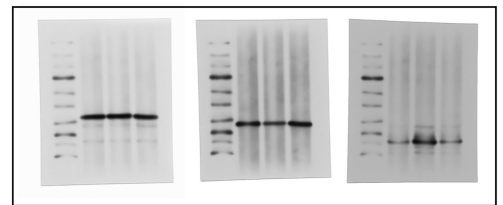

**F**

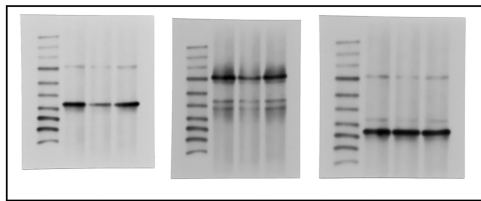

**G**

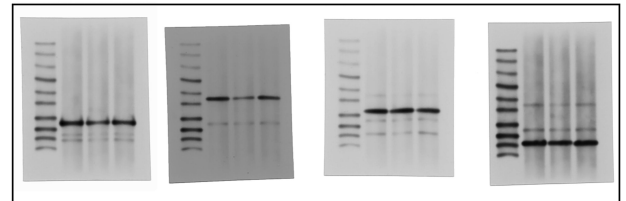

**H**

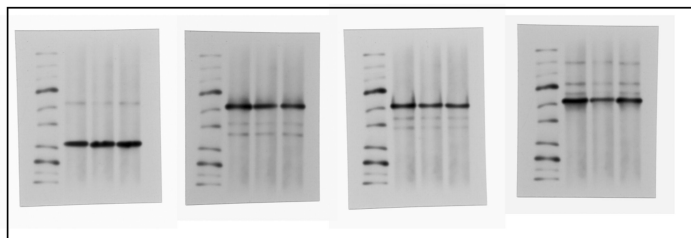

**I**

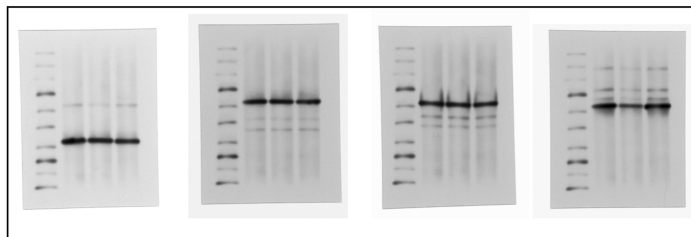

**J**

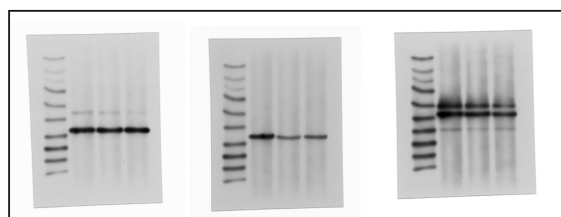

**K**

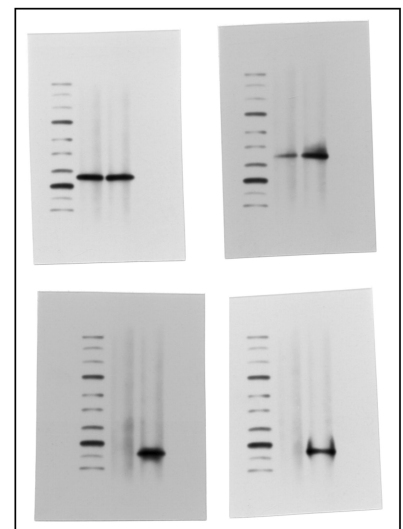

**L**

Supplement: Supplementary file 1 — Supplementary Material [file 13287_2025_4276_MOESM1_ESM.pdf]
